# Closed-Pore Formation in Oxygen Electrodes for Solid Oxide Electrolysis Cells Investigated by Impedance Spectroscopy

**DOI:** 10.1021/acsami.2c20731

**Published:** 2023-02-02

**Authors:** Martin Krammer, Alexander Schmid, Andreas Nenning, Andreas Ewald Bumberger, Matthäus Siebenhofer, Christopher Herzig, Andreas Limbeck, Christoph Rameshan, Markus Kubicek, Juergen Fleig

**Affiliations:** † Institute of Chemical Technologies and Analytics, Technische Universität (TU) Wien, Getreidemarkt 9/164-EC, 1060Vienna, Austria; ‡ Centre for Electrochemical Surface Technology GmbH, Viktor-Kaplan-Straße 2, 2700Wiener Neustadt, Austria; ¶ Institute of Material Chemistry, Technische Universität (TU) Wien, Getreidemarkt 9/165-PC, 1060Vienna, Austria; § Chair of Physical Chemistry, Montanuniversität Leoben, Franz-Josef-Straße 18, 8700Leoben, Austria

**Keywords:** solid oxide electrolysis cell (SOEC), oxygen electrode, degradation mechanism, pore formation, impedance spectroscopy, chemical capacitance, La_0.6_Sr_0.4_CoO_3−δ_ (LSC), thin film

## Abstract

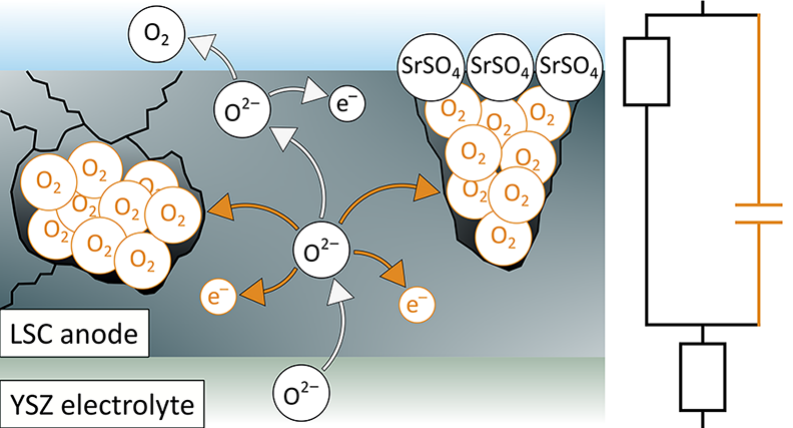

Electrochemical impedance spectroscopy was used to investigate the chemical capacitance of La_0.6_Sr_0.4_CoO_3−δ_ (LSC) thin-film electrodes under anodic polarization (i.e., in the electrolysis mode). For this purpose, electrodes with different microstructures were prepared via pulsed-laser deposition. Analysis of dense electrodes and electrodes with open porosity revealed decreasing chemical capacitances with increasing anodic overpotentials, as expected from defect chemical considerations. However, extremely high chemical capacitance peaks with values in the range of 10^4^ F/cm^3^ at overpotentials of >140 mV were obtained after annealing for several hours in synthetic air and/or after applying high anodic bias voltages of >750 mV. From the results of several surface analysis techniques and transmission electron microscopy, it is concluded that closed pores develop upon both of these treatments: (i) During annealing, initially open pores get closed by SrSO_4_, which forms due to strontium segregation in measurement gases with minute traces of sulfur. (ii) The bias treatment causes mechanical failure and morphological changes including closed pores in the bulk of dense films. Under anodic polarization, high-pressure oxygen accumulates in those closed pores, and this causes the capacitance peak. Model calculations based on a real-gas equation allow us to properly predict the experimentally obtained capacitance increase. We demonstrate that analysis of the chemical capacitance of oxygen electrodes in solid oxide electrolysis cells can thus be used as a nondestructive observation tool to detect and quantify closed porosity with a lower detection limit between 10^–4^ and 10^–3^.

## 1 Introduction

Solid oxide electrolysis cells (SOECs) have the potential of playing an important role for several industrial sectors because they enable highly efficient production of hydrogen, CO, or syngas from electrical energy.^1−4^ For example, SOECs may transform excess electrical energy from renewables to hydrogen, which could then be used for ammonia and steel production, as fuel in the mobility sector or for converting it back to electrical energy to cope with supply and demand issues.^5,6^ However, stability problems and performance deterioration are still major challenges for this technology.^7−14^ Many of these issues are related to the oxygen electrode (anode) and/or the oxygen electrode/electrolyte interface. Typically, the oxygen electrode consists of a perovskite-type oxide such as La_1–*x*_Sr_*x*_MnO_3−δ_ (LSM) or La_1–*x*_Sr_*x*_Co_*y*_Fe_1–*y*_O_3−δ_ (LSCF), and Y_2_O_3_-doped ZrO_2_ (YSZ) is most commonly used as the electrolyte. Some of the reported degradation phenomena are related to the formation of secondary phases, e.g., La_2_Zr_2_O_7_ or SrZrO_3_ at the oxygen electrode/electrolyte interface^15−17^ or SrO and SrCrO_4_ on the electrode surface,^17^ as well as sulfur poisoning.^18−20^

Other studies revealed problems of mechanical nature under SOEC operation, including the development of pores and cracks in the electrolyte^14,15^ or at the oxygen electrode/electrolyte interface^13,21,22^ and even delamination of the oxygen electrode from the electrolyte or the barrier layer.^10,15,16,21,23−26^ This kind of degradation is suggested to be caused by high internal gas pressures, leading to mechanical stress.^10,21,24,26−28^ Hence, it would be beneficial to observe the buildup of internal gas pressures at an early stage in order to prevent destructive mechanical load. In a recent study,^29^ it was shown that thin-film electrodes with an intentionally built-in closed porosity exhibit a capacitance maximum at moderate anodic polarization. Thermodynamic calculations based on a real-gas model clearly demonstrate that the buildup of high-pressure oxygen in closed pores causes this chemical capacitance peak. These findings also indicate that such capacitance measurements might be a highly sensitive tool for detecting closed pores in or near oxygen electrodes of SOECs.

In this study, we apply this tool to investigate the chemical capacitance of La_0.6_Sr_0.4_CoO_3−δ_ (LSC) thin-film electrodes after annealing and bias voltage treatments, which are both known to induce degradation phenomena in oxygen electrodes of SOECs. We observed that electrodes with initially open pores or cracks exhibit a capacitance peak under anodic polarization (i.e., in the electrolysis mode) after several hours of annealing in synthetic air. A peak of the chemical capacitance also develops in the case of dense electrodes after the application of high anodic bias voltages of >750 mV. It is shown that in both cases degradation mechanisms lead to the formation of closed pores, and those can be detected by the chemical capacitance. The quantitative model from ref (29) is used to estimate the amount of closed porosity and indicates the potential of the presented electrochemical method for detecting and quantifying closed pores in or near oxygen electrodes of SOECs.

## 2 Experimental Section

### 2.1 Sample Preparation

**Figure 1:**
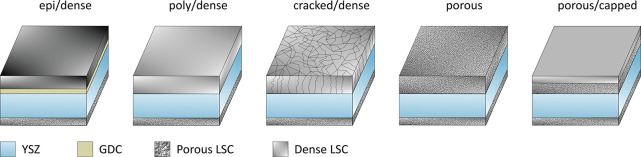
Sketches and nomenclature of the different sample types investigated in this study.

All thin films of this study were deposited on yttria-stabilized zirconia (YSZ) single crystals [5 × 5 × 0.5 mm^3^, (100)-oriented, 9.5 mol % Y_2_O_3_; CrysTec, Germany], which served as electrolytes and substrates. The thin-film working and counter electrodes were prepared via pulsed-laser deposition (PLD). The targets for PLD of La_0.6_Sr_0.4_CoO_3−δ_ (LSC) were fabricated by Pechini synthesis, and the obtained powder was calcined for 10 h at 800 °C. Thereafter, the powder was pressed into a pellet by cold isostatic pressing (300–310 MPa), followed by a sintering step of 12 h at 1200 °C in air. Film deposition was performed in a vacuum chamber with a KrF excimer laser (248 nm; Compex Pro 201F, Coherent, Germany). First, porous LSC counter electrodes were prepared by deposition at a substrate temperature of 450 °C and at an oxygen partial pressure of 0.4 mbar. These parameters were chosen based on former studies,^30,31^ which showed that LSC electrodes fabricated under these conditions exhibit very low polarization resistances due to nanopores, leading to an increased surface area. Afterward, the LSC working electrodes were deposited. By varying the deposition parameters, five sample types were fabricated, which are different in terms of the microstructure (i.e., porosity and surface area) and the crystal structure of the working electrode: (i) highly oriented, resembling epitaxial dense (epi/dense), (ii) polycrystalline dense (poly/dense), (iii) polycrystalline with cracks, otherwise dense (cracked/dense), (iv) porous, and (v) porous films with a dense capping layer on top (porous/capped) (Figure 1). The deposition parameters for the individual sample types are shown in Table 1.

**Table 1:** Deposition Parameters for the Five Different Sample Types Investigated in This Study

sample type	temperature (°C)	oxygen partial pressure (mbar)	target–substrate distance (cm)	special treatment
epi/dense	600	0.04	6	GDC interlayer
poly/dense	600	0.04	6	
cracked/dense	600	0.04	6	deposited before the counter electrode
porous	450	0.4	5	
porous/capped	450/600	0.4/0.04	5/6	poly/dense film on top

Shortly before each deposition, the substrate temperature was determined with a pyrometer, which was tuned to the emissivity of YSZ. The laser fluence inside the vacuum chamber was adjusted to approximately 1.1 J/cm^2^ for the deposition of dense films (epi/dense, poly/dense, and cracked/dense) and to about 1.4 J/cm^2^ for the deposition of porous films. Thus, all films exhibited similar compositions with an average of La_0.605±0.023_Sr_0.407±0.014_Co_0.989±0.019_O_3−δ_ determined by inductively coupled plasma mass spectroscopy (ICP-MS). The laser was operated with a pulse repetition rate of 5 Hz for all depositions. For preparation of the epi/dense films, 100 laser shots were fired onto a Ce_0.8_Gd_0.2_O_2−δ_ (GDC) target prior to deposition of the dense LSC film, yielding a GDC interlayer of about 6 nm thickness. The parameters for deposition of the poly/dense films were taken from former studies,^29,32,33^ in which cross sections from transmission electron microscopy (TEM) revealed densely packed columnar growth. The cracked/dense films were produced by changing the routine of the fabrication process: The film of the working electrode was deposited before the counter electrode preparation, i.e., before any heat exposure of the YSZ single crystal. Following this routine, cracks formed in the working electrode (Figure 3c). The deposition parameters for the porous samples were also chosen according to previous studies, which used bright-field TEM (BF-TEM) cross sections, high-angle annular dark field (HAADF), and ICP-MS measurements in order to demonstrate the porosity of the respective films.^29,30^ The porous/capped electrodes were finally produced by first depositing a standard porous film, followed by an immediate change of the deposition parameters in order to deposit a dense capping layer with a thickness ranging from 8 to 15 nm. This deposition routine was also reported in a recent study,^29^ in which TEM cross sections confirmed the stacking of a dense LSC layer on top of a porous one. The porous part of those electrodes amounted to about 80% of the total electrode thickness. Right after deposition, all samples were cooled in their respective atmospheres with a cooling rate of 15 °C/min. Film thicknesses were determined from ICP-MS measurements using the lattice parameter from X-ray diffraction (XRD) and with a profilometer (DektakXT, Bruker, USA), respectively. By variation of the amount of pulses, total electrode thicknesses between 43 and 285 nm were obtained.

The LSC films fabricated as working electrodes were then microstructured via photolithography and ion-beam etching. In the first step of the photolithography process, the samples were coated with a photoresist (ma-N 1420 MicroResist Technology, Germany) while spinning on a spin-coater. Afterward, the samples were placed on a heating stage for 5 min at 100 °C in order to evaporate the excess solvent. In the next step, a patterned shadow mask was placed above the sample, and UV light (350 W, USHIO 350DP Hg, Japan) was employed to transfer the shape of circular microelectrodes with diameters of 195–300 μm to the photoresist. A different mask was used for the electrodes prepared for additional near-ambient-pressure X-ray photoelectron spectroscopy (NAP-XPS) measurements, which yielded rectangular electrodes with an area of 0.41 mm^2^. The parts of the photoresist not being illuminated were removed with a developer solution (ma-D 533/s, MicroResist Technology, Germany). The uncovered areas of the films were etched away with an ion beam (KDC 40, Kaufman & Robinson, USA). For this purpose, a diffuse argon plasma was operated at 9 × 10^–4^ mbar of argon with a beam voltage of 500 V and a beam current of 10 mA. The remaining photoresist on top of the microelectrodes was carefully removed using a clean room wipe soaked in ethanol.

### 2.2 Impedance Spectroscopy

For most of the measurements, the samples were placed inside a closed fused-silica apparatus and symmetrically heated in a tube furnace to temperatures between 450 and 610 °C. A type S thermocouple located within 1 cm of the sample was used to measure the temperature. The samples were placed on a platinum mesh to ensure electrical contact of the counter electrodes. For electrical contact of the (working) microelectrodes, platinum–rhodium needles were applied and positioned with a microscope camera. Only the experiments with cracked/dense LSC thin films were conducted with a different measurement setup. Those samples were placed in a vacuum chamber onto a corundum heating stage, which was coated with platinum in order to apply electrical contact of the counter electrodes. The (working) microelectrodes were contacted with a platinum needle, again using a microscope camera. Because of the asymmetric heating in this measurement setup, the temperature of the working electrodes was calculated from the high-frequency *x*-axis intercept in the impedance spectra, which corresponds to the ionic transport resistance *R*_YSZ_ of the electrolyte.^34,35^

Impedance spectra were recorded with direct-current (DC) bias voltages ranging from 0 to 1000 mV using an Alpha-A high-performance frequency analyzer and an Electrochemical Test Station POT/GAL 30 V/2 A (both from Novocontrol, Germany). All impedance measurements were conducted with an alternating root-mean-square voltage of 10 mV in the frequency range of 10^6^–10^–2^ Hz with 5 data points/decade. The Electrochemical Test Station was also used to measure the DC voltages and currents. Most of the measurements were performed in synthetic air. For the *in situ* NAP-XPS experiments and corresponding *ex situ* measurements, which were carried out for the purpose of comparison, an oxygen/nitrogen mixture with an oxygen partial pressure of 1 mbar was used. High-purity gases (99.999%, Messer Austria GmbH, Austria) were employed for all experiments.

### 2.3 Structural Characterization

XRD measurements were performed to investigate the crystal structures of the different thin films and microelectrodes using an Empyrean X-ray diffractometer (Malvern Panalytical, U.K.) with a copper radiation source in grazing-incidence and Bragg–Brentano geometry. For the grazing-incidence scans, which were performed at an incidence angle of 2 °, a parallel beam mirror on the incident-beam side and a parallel-plate collimator, together with a scintillation detector, on the diffracted-beam side were employed. A focusing mirror on the incident-beam side and a semiconductor area detector (GaliPix3D, Malvern Panalytical, U.K.) on the diffracted-beam side were applied for the measurements in Bragg–Brentano geometry. In order to focus the beam onto individual microelectrodes, a 0.3 mm slit was used.

Atomic force microscopy (AFM) measurements were conducted in order to analyze the surface structures of different LSC samples [Nanoscope V multimode setup (Bruker, USA) operated in tapping mode].

### 2.4 ICP-MS

The elemental composition of the LSC films was determined via an inductively coupled plasma mass spectrometer, equipped with a quadrupole mass filter and a collision cell (iCAP QC, Thermo Fisher Scientific, Germany). Before the actual analysis, a two-step dissolution was performed according to former studies:^29,33^ At first, the water-soluble strontium species, which possibly formed on the surface of the LSC thin films, were dissolved in 5 mL of freshly prepared ultrapure water (BarnsteadTM EasypureTM II, 18.2 MΩ cm) for 30 min. In the second step, 100 μL of concentrated HCl was used to completely dissolve the remaining LSC thin film. The obtained data were processed using *Qtegra* software (Thermo Fisher Scientific, USA). Further details regarding the ICP-MS measurements were reported in a previous paper.^29^

### 2.5 *In Situ* NAP-XPS

NAP-XPS measurements were performed while simultaneously recording electrochemical impedance spectra as described above. NAP-XPS was carried out in a laboratory-based machine with monochromated Al Kα radiation (μ FOCUS 500 NAP, SPECS, Germany) at an oxygen pressure of 1 mbar. The instrument was further equipped with a differentially pumped hemispherical electron energy analyzer (PHOIBOS 150 NAP, SPECS, Germany), which had a water-cooled nozzle and a sample stage optimized for solid-state electrochemical characterization. Details regarding the sample stage can be found in an earlier study.^36^ The sample was mounted on a platinum-coated Al_2_O_3_ disk with a 4.5 × 4.5 mm^2^ central bore on which the sample with a size of 5 × 5 mm^2^ was located. This enabled direct sample heating with the near-infrared laser, as sketched in Figure S4. The macroscopic porous LSC counter electrode was contacted through the sample stage, and the specifically designed rectangular LSC electrode (380 × 1080 μm^2^) was contacted by a platinum–iridium tip. In order to avoid XPS peak shifts due to the applied voltage, the microelectrode was grounded and a negative bias was applied to the counter electrode.

The temperature of the sample was controlled using the high-frequency *x*-axis intercept in the impedance spectra, as described above. Due to the rectangular electrode geometry and a contribution of the electrode sheet resistance, the relationship of the sample temperature and high-frequency resistance was calibrated *ex situ* in the homogeneously heated apparatus prior to the NAP-XPS measurements. XPS spectra were collected at an analyzer pass energy of 30 eV and processed by the software *CasaXPS*. An S-shaped “Shirley” background was used for all peaks. Components were fitted primarily with mixed Gaussian–Lorentzian peak shapes (for Sr 3d, most O 1s components, and S 2p). Additionally, an asymmetric component (“LF” peak shape in *CasaXPS*) was used for the second O 1s “bulk” component. Spin–orbit doublets of the p and d orbitals were constrained to have equal full width at half-maximum, appropriate area ratio (2:3 for d and 1:2 for p transitions), and fixed energy separation (1.6 eV for Sr 3d and 1.2 eV for S 2p). For chemical quantification, the peak areas were further corrected for the photoexcitation cross section,^37^ analyzer transmission function, and energy dependence of the photoelectron inelastic mean-free path (IMFP ≈ *E*_kin_^0.8^).

### 2.6 TEM

For TEM investigations, an electron-transparent lamella was prepared via standard lift-out techniques with a focused ion beam/scanning electron microscopy system (Scios 2 DualBeam, Thermo Fisher Scientific, Germany), operating with a gallium-ion beam at 30 kV accelerating voltage. Final low-voltage cleaning of the lamella was performed at 2 and 5 kV. All TEM measurements were carried out on a 200 kV FEI TECNAI F20 microscope equipped with an EDAX APOLLO XII detector for energy-dispersive X-ray spectroscopy (EDX). Scanning transmission electron microscopy (STEM) and EDX line scans were performed with a probe size of approximately 2 nm and HAADF camera lengths of 350 and 490 mm.

## 3 Structural and Electrochemical Characterization

### 3.1 XRD

**Figure 2:**
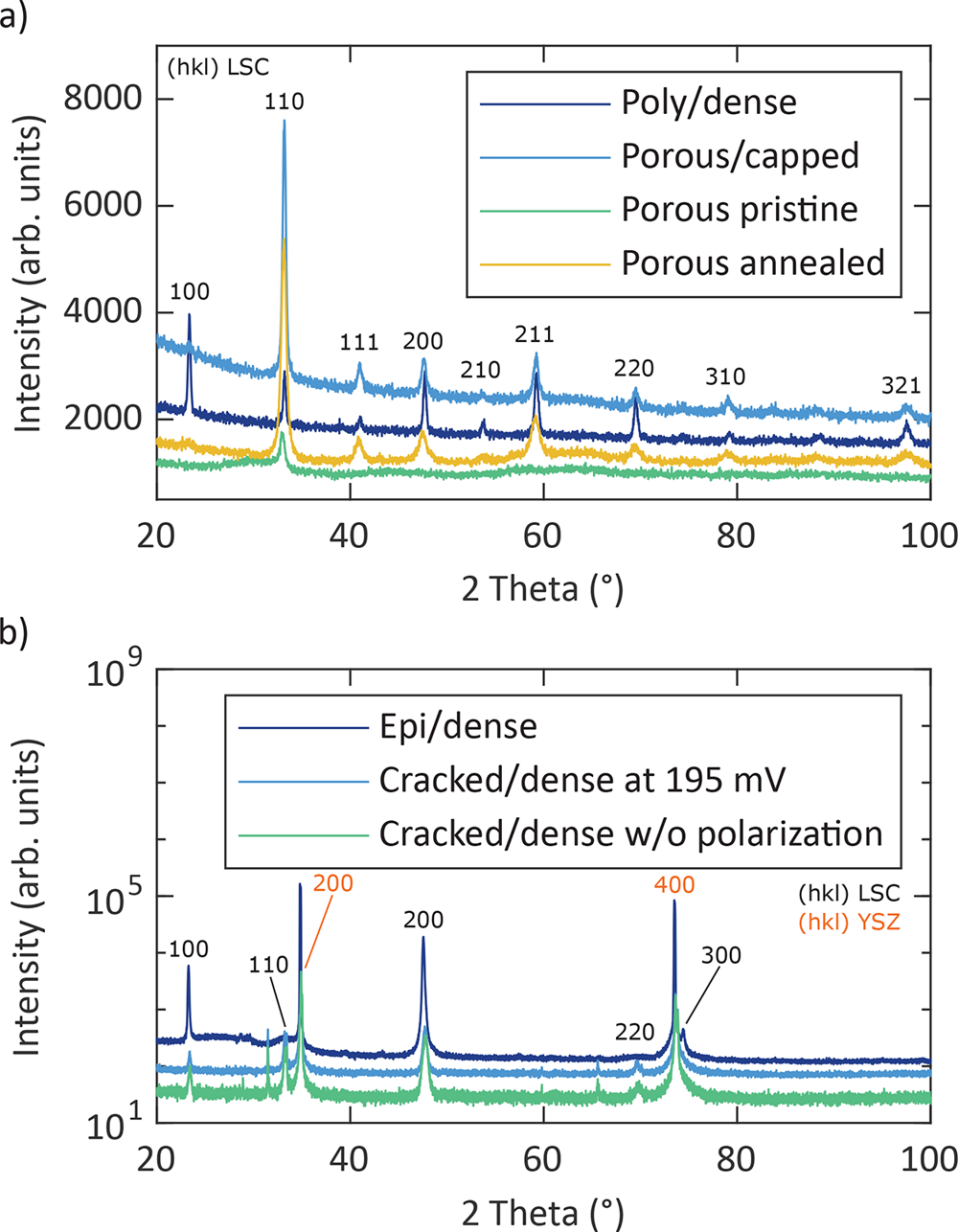
XRD diffractograms of pristine poly/dense,^29^ pristine porous/capped,^29^ pristine,^29^ and annealed porous films measured in the grazing-incidence geometry (a) as well as of epi/dense and cracked/dense (“frozen” at 195 mV and 600 °C and without polarization history at 600 °C) films measured in the Bragg–Brentano geometry (b).

XRD measurements of the different sample types showed that all reflexes measured in the grazing-incidence and Bragg–Brentano geometry could be assigned to either the pseudocubic structure of the LSC perovskite phase or the YSZ phase of the substrate (Figure 2). The intensities of the individual reflexes varied between the different sample types and the applied measurement geometries. Diffractograms of pristine poly/dense, pristine porous, and pristine porous/capped films were also part of an earlier study.^29^Figure 2a shows that the pristine porous film exhibits a low degree of crystallinity because only one distinct peak is visible. However, after annealing for 54 h at 608 °C, the porous LSC film shows all of the reflexes obtained for the poly/dense film. Thus, some postcrystallization seems to occur in porous films upon annealing. The diffractogram of the epi/dense film, which was measured in the Bragg–Brentano geometry, suggests highly oriented growth resembling fully epitaxial films because only peaks from the (100) planes were found (Figure 2b). Moreover, Figure 2b shows diffractograms of a cracked/dense microelectrode “frozen” at 195 mV overpotential and 600 °C as well as of a cracked/dense microelectrode “frozen” at 600 °C without polarization. The “frozen” samples were removed from the heating stage (at 600 °C) during an impedance measurement with/without DC bias voltage and rapidly cooled to room temperature prior to the XRD measurement. No significant differences between the diffraction patterns of these two cracked/dense electrodes were observed. The low intensity of the corresponding peaks resulted from the application of a narrow slit, which was used to focus the beam onto individual microelectrodes. The unlabeled peaks at about 31.5° and 65.5° resulted from a reflection corresponding to the Cu Kβ wavelength of the radiation source.

### 3.2 AFM

**Figure 3:**
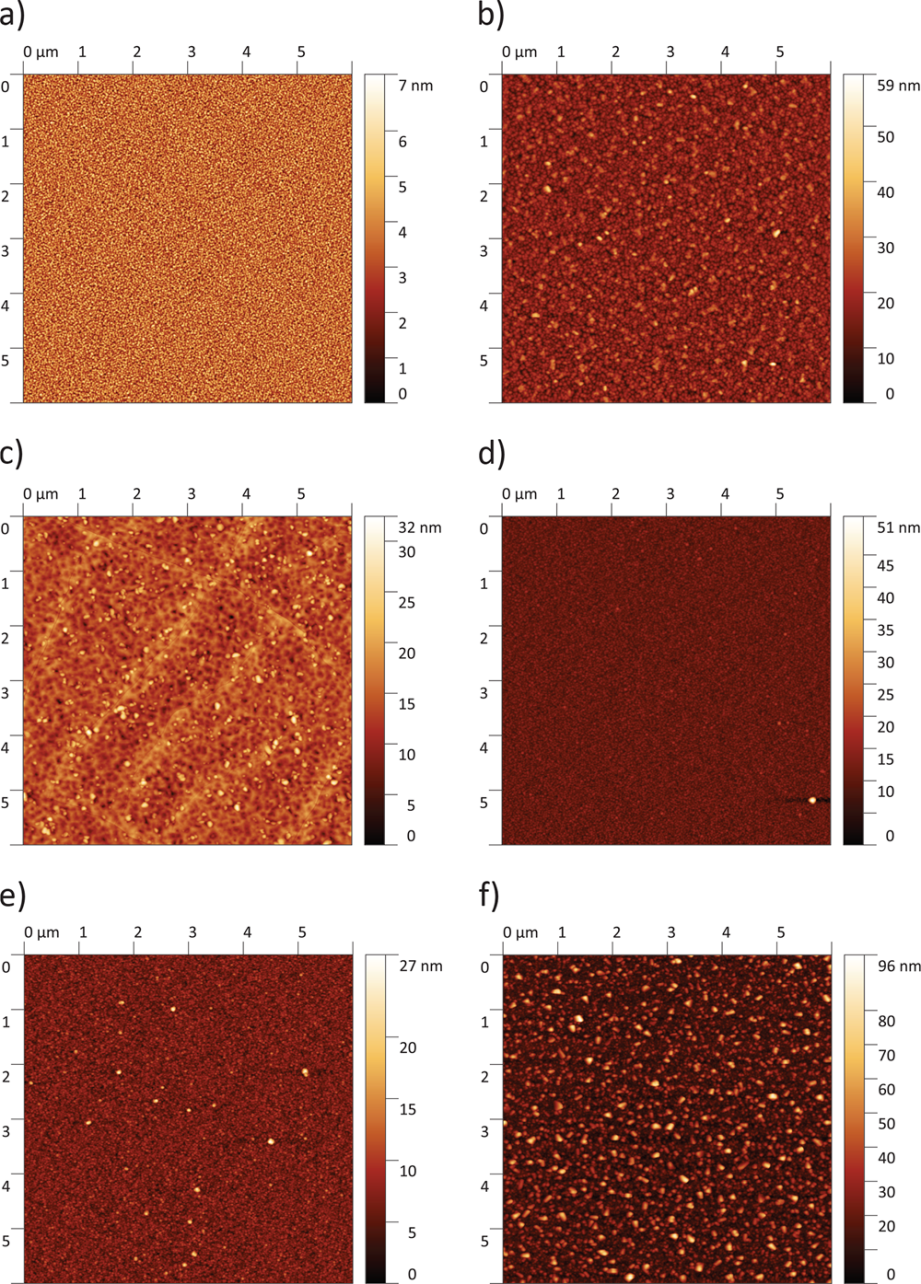
AFM scans of the different LSC sample types investigated in this study: pristine epi/dense (a), pristine poly/dense (b), pristine cracked/dense (c), pristine porous/capped (d), pristine porous (e), and annealed porous (f).

Figure 3 displays AFM scans of pristine films corresponding to the different sample types investigated in this study. All films show distinguishable and homogeneously distributed grains. Films without a GDC interlayer, which were deposited at 600 °C and 0.04 mbar (poly/dense and cracked/dense), exhibit a larger grain size than those deposited at 460 °C and 0.4 mbar (porous and porous/capped), as expected from earlier studies.^32,38^ The surface of the porous/capped film shows a grain size similar to that of the porous film, despite the deposition of a dense capping layer at 600 °C and 0.04 mbar. Hence, it is assumed that this dense capping layer adapts its grain size to the porous layer underneath. The AFM image of the cracked/dense sample also displays the cracks that were distinctive for these films. Furthermore, a porous film is shown that was annealed for more than 100 h at 608 °C. Through a comparison of the pristine and annealed porous film, it becomes apparent that large grains with heights of up to about 100 nm were formed during annealing.

### 3.3 Impedance Spectroscopy

**Figure 4:**
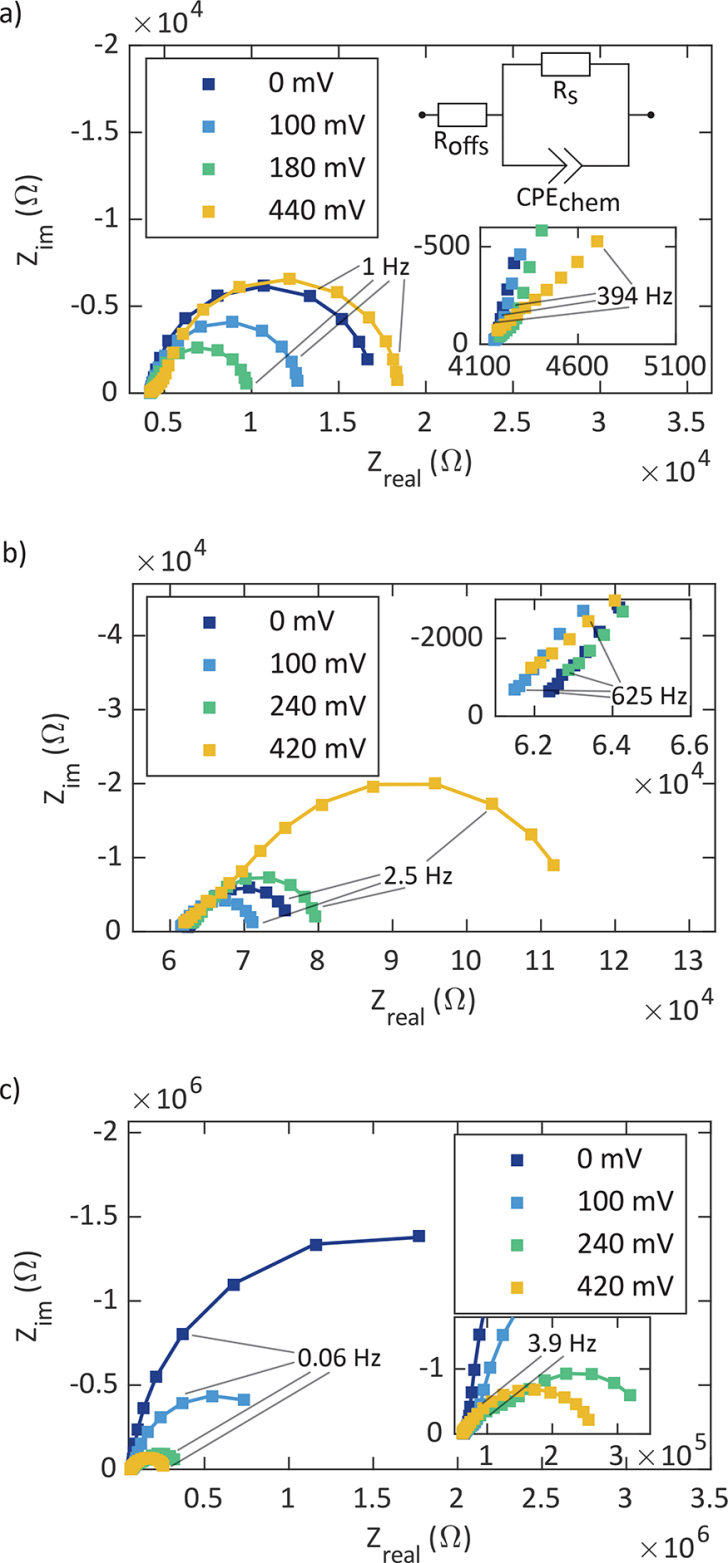
Impedance spectra of a pristine poly/dense (a), a pristine porous (b) and an annealed (6 h at 460 °C) porous (c) LSC thin film microelectrode at various DC bias voltages (*U*_DC_) at measurement temperatures of 608 °C (poly/dense) and 460 °C (porous). Solid lines are fits to the equivalent circuit shown in part a (with the exception of spectra at 420 mV in parts b and c; see section 2.2).

The exemplary impedance spectra in Figure 4 show data from impedance measurements with applied bias voltages ranging from 0 to 440 mV.

Note that the spectra of epi/dense electrodes are very similar to those shown for poly/dense electrodes (Figure 4a). Furthermore, porous/capped electrodes exhibit spectra similar to those for annealed porous electrodes (Figure 4c). All spectra contain a high-frequency *x*-axis intercept, which is temperature-dependent but independent of the applied bias voltage. In accordance with the literature,^30,34,39^ this intercept can be attributed to the ionic transport resistance of the YSZ electrolyte (*R*_YSZ_). The value of *R*_YSZ_ was determined from the intersection of the *x* axis with extrapolation of the electrode-related impedance feature. At intermediate frequencies, features that vary depending on the sample type and on the annealing time and generally become larger with increasing bias voltage are visible. Such contributions were also reported in previous studies on LSC^30,32,33^ and are often associated with interfacial processes between the electrolyte and the electrode. As can be seen in Figure 4, some spectra resemble a Warburg-like behavior in the intermediate frequency range, which may indicate the onset of an oxygen diffusion limitation in the LSC working electrode. However, a finite Warburg element alone never yielded reasonable fit results of these spectra. Given that these intermediate frequency features were not the focal point of this study, their contributions were not examined in more detail.

At low frequencies, all spectra contain a semicircular-type feature, which is associated with the oxygen surface exchange resistance *R*_s_ and the chemical capacitance *C*_chem_ of the working electrode, in agreement with former studies on LSC and similar mixed conducting oxides.^30,33,38−41^ Evaluation of the chemical capacitance (*C*_chem_) under anodic polarization was the main focus of this work. For this purpose, the low-frequency semicircle was fitted to a parallel connection of a constant-phase element (CPE_chem_) and a resistance (*R*_s_) using a nonlinear least-squares method. The impedance of a constant-phase element is defined as
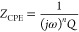
1which considers the nonideal behavior of a capacitance. With the parameter *Q* and exponent *n*, both obtained from the fitting procedure, *C*_chem_ can be calculated via^42^
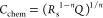
2Apart from the parallel connection of CPE_chem_ and *R*_s_, the equivalent circuit used for the fitting procedure consisted of an offset serial resistance *R*_offs_ (see the circuit in Figure 4a). This *R*_offs_ considers the above-described impedance contributions from the electrolyte (*R*_YSZ_) and any intermediate frequency features. This simple equivalent circuit yielded the most reliable determination of *C*_chem_, as long as the low-frequency semicircle accounted for the major part of the corresponding spectrum and was reasonably well separated from the intermediate-frequency features. Under these conditions, the oxygen chemical potential in the whole working electrode (μ_O_^WE^) was determined by the electrode’s overpotential η_WE_ according to
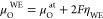
3The symbol μ_O_^at^ represents the oxygen chemical potential in the gas phase, and η_WE_ was calculated via
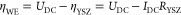
4Here, *U*_DC_ is the applied DC bias voltage, *I*_DC_ stands for the DC current, and η_YSZ_ is the electrolyte’s overpotential caused by the finite ionic conductivity of YSZ. Accordingly, deviations caused by any interfacial resistance or transport limitation in the electrode were neglected. Also, any overpotential contribution from the counter electrode was neglected because its active area was at least 350 times larger than that of the (working) microelectrodes and its polarization resistance was very low, as shown in a former study.^30^

In most cases, the above-described conditions were reasonably met; however, at very high bias voltages, some spectra revealed intermediate-frequency contributions sized similarly to the low-frequency feature and partly merged with the latter. Such spectra (e.g., spectra at 420 mV in Figure 4b,c) would require a more sophisticated impedance analysis (at least two *R*/CPE elements and a resistance in series) and are not included in the following chemical capacitance analysis.

In general, oxygen exchange rates are governed by the concentrations of defects (holes and oxygen vacancies),^43,44^ which, in turn, are strongly dependent on the overpotential.^41^ Consequently, a significantly bias-dependent surface exchange resistance *R*_s_ can be expected. In combination with the nontrivial defect chemistry of LSC^45−47^ and possible irreversible changes at very high anodic overpotentials (see below), the overpotential dependence of *R*_s_ is supposed to be rather complex. Because the chemical capacitance rather than the surface exchange resistance was the main focus of this study, we did not investigate this overpotential-dependent behavior in detail. For the sake of completeness, Figure S1 shows the *R*_s_ values of pristine and annealed electrodes of all different sample types determined with the simple equivalent circuit depicted in Figure 4a.

## 4 Chemical Capacitance Analysis

### 4.1 Chemical Capacitance of Pristine Electrodes

**Figure 5:**
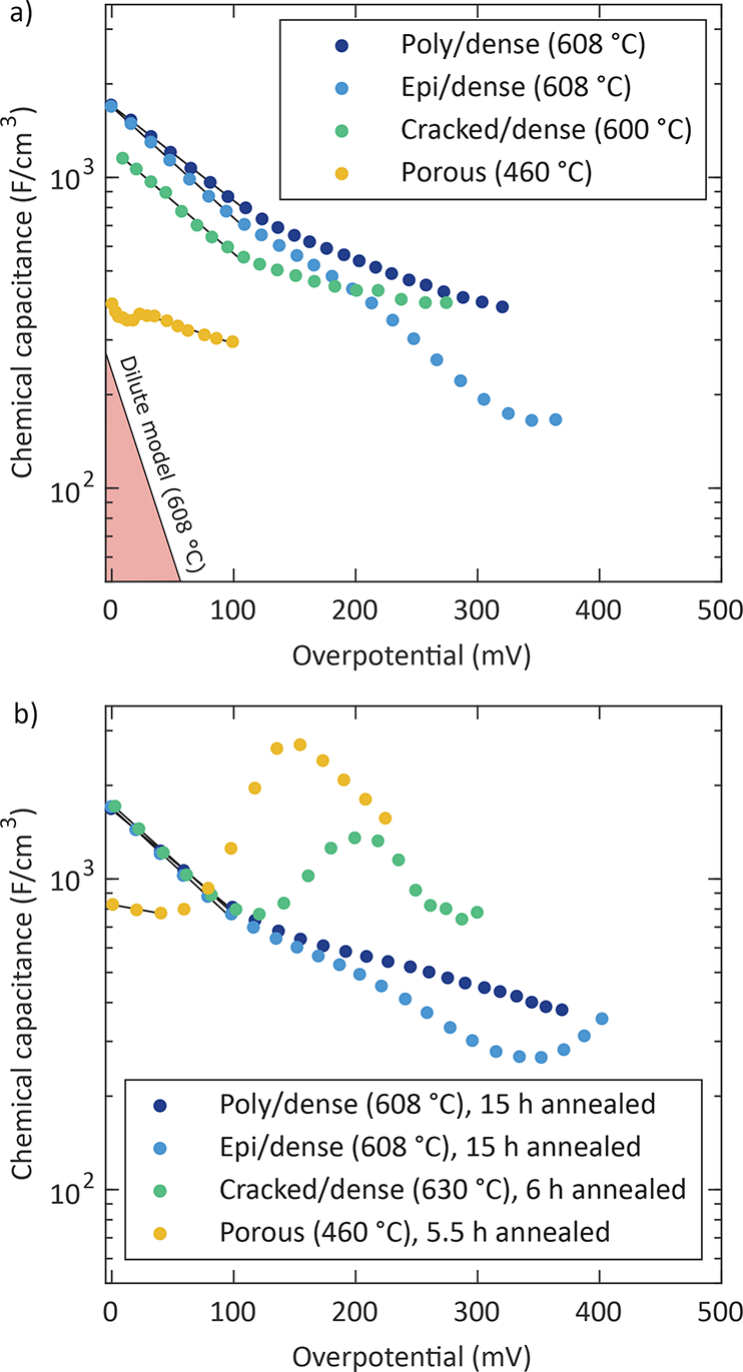
Chemical capacitance of different pristine (a) and annealed (b) LSC electrodes as a function of the electrode overpotential, measured at the indicated temperature and corresponding fits (solid line). The red triangle represents the slope corresponding to the dilute defect model at 608 °C.

Figure 5a displays the chemical capacitance curves of pristine electrodes as a function of the electrode overpotential. In this and all subsequent figures, the chemical capacitance values were normalized to the respective entire electrode volume, without subtracting the cavity and pore volumes of the cracked/dense, porous, and porous/capped electrodes. The poly/dense and the epi/dense electrodes were measured at 608 °C (thermocouple), whereas a temperature of 600 °C was determined from *R*_YSZ_ for the cracked/dense electrode. Due to the superior oxygen exchange kinetics of pristine porous electrodes (*R*_s_ < 0.1 Ω cm^2^ at 608 °C in synthetic air), their chemical capacitance was analyzed at a lower temperature (i.e., 460 °C) in order to obtain data at overpotentials comparable to the measurements on the other electrodes. Nevertheless, the chemical capacitance could only be evaluated up to η_WE_ ≈ 100 mV because of a strong merging of the intermediate- and low-frequency features.

The curves of all pristine electrodes reveal a decrease of the chemical capacitance with increasing anodic overpotential (Figure 5a). This behavior is consistent with previous studies on LSC and similar mixed conducting oxides.^29,39−41^ The porous/capped electrode shows a different behavior and will be discussed later in this section. The chemical capacitance is usually defined as follows:^48,49^
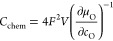
5with *F* denoting the Faraday constant. Hence, *C*_chem_ scales with the electrode’s volume *V* and the inverse derivative of the oxygen chemical potential μ_O_ with respect to the oxygen concentration *c*_O_. Assuming dilute defects in an acceptor-doped mixed conducting oxide, the chemical capacitance of the solid can be expressed in terms of the concentrations of electronic defects (*c*_eon_) and oxygen vacancies (*c*_V_) as
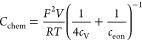
6*T* and *R* denote the temperature and universal gas constant, respectively. Obviously, the chemical capacitance is largely determined by the minority charge carriers, which are oxygen vacancies in many SOEC-relevant perovskites under these conditions. In order to analyze the voltage-dependent chemical capacitances properly, it is useful to consider the individual contributions of atmosphere and voltage to the oxygen chemical potential inside the working electrode. Relative to 1 bar of oxygen, it is given by^40,41^
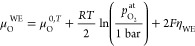
7where  stands for the actual atmospheric oxygen partial pressure and μ_O_^0,*T*^ is the chemical potential of oxygen gas at 1 bar. Note that eq 7 is valid as long as the transport of charge carriers in the electrode is fast in comparison to the oxygen exchange reaction at the surface and the atmospheric oxygen partial pressure  reasonably well approximates the respective oxygen fugacity  (see below). Moreover, we neglect that a part of the electrode overpotential η_WE_ refers to intermediate-frequency features, which do not alter μ_O_^WE^ in the entire film. However, given that *C*_chem_ was only evaluated for spectra with dominating low-frequency arcs, eq 7 represents a solid approximation.

Accordingly, both increasing (anodic) overpotential and increasing oxygen partial pressure lead to an increase of the oxygen chemical potential in the working electrode, as was also experimentally demonstrated in former studies.^40,41^ Thus, we may define an effective internal oxygen partial pressure within the oxide of the working electrode (40,41,50) by
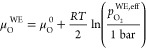
8and together with eq 7, one gets
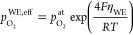
9In accordance with defect chemical studies on LSCF in the literature,^40,41,45,46,51−55^ we attribute the measured decrease of the chemical capacitance values under increasing anodic overpotential to the decrease of the oxygen vacancy concentration. From the dilute defect model of an acceptor-doped oxide, we get
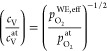
10provided that the atmospheric vacancy concentration *c*_V_^at^ is already in the range where vacancies are minority charge carriers. Combining eqs 10, 9, and 6 leads to
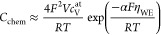
11with an exponential factor α = 2 (see the bottom left corner in Figure 5a). For the poly/dense, epi/dense, and cracked/dense electrodes, fits of up to 100  mV yielded exponential factors between 0.5 and 0.6. This is close to the value obtained by Kawada et al.^40^ (0.7) for La_0.6_Sr_0.4_CoO_3−δ_ electrodes deposited on a Ce_0.9_Ca_0.1_O_1.95_ electrolyte substrate and measured at 600 °C and 0.1 bar of oxygen partial pressure. The data for the pristine porous electrode do not follow a linear slope even at low overpotentials, which may be caused by crystallization effects because postcrystallization was observed in the corresponding XRD measurements (Figure 2a). However, deviations from the dilute defect model are not surprising considering the metal-like character of LSC,^45,46^ and we still assume that the chemical capacitances in Figure 5a are all determined by the overpotential-dependent oxygen vacancy concentrations.

**Figure 6:**
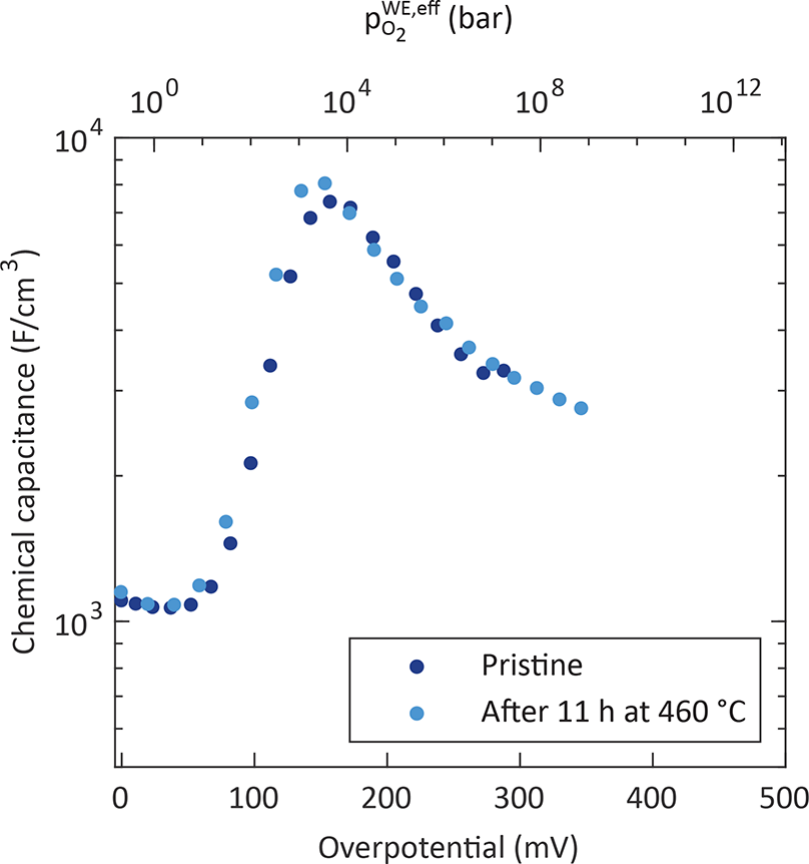
Chemical capacitance of pristine and annealed (11 h at 460 °C) porous/capped electrodes as a function of the electrode overpotential and the corresponding effective internal oxygen partial pressure, respectively, measured at 460 °C.

Figure 6 reveals that pristine porous electrodes with a polycrystalline, supposedly dense capping layer exhibit a completely different behavior compared to the pristine electrodes of all other sample types: After an initial minor decrease of the chemical capacitance at low overpotentials, a very pronounced peak can be observed with a maximum of >8000 F/cm^3^ at about 150 mV. At overpotentials of >150 mV, the capacitance decreases again. This peak-shaped curve cannot be explained by the standard defect chemical interpretation of the chemical capacitance in an oxide.^39−41^ Instead, another redox reaction has to be involved. This phenomenon was discussed and interpreted in detail in a previous work.^29^ There, it was shown that the formation of highly pressurized oxygen in closed pores causes this capacitive effect. Because overpotentials between 150 and 250 mV would correspond to  values ranging from 2.8 × 10^3^ to 1.6 × 10^6^ bar according to eq 9, it is necessary to consider a real-gas equation to predict the experimentally obtained peak-shaped capacitance curves^29^ (see also below). Consequently, a relationship like that in eq 9 cannot be used for describing the gas pressure in closed pores; instead, it corresponds to the fugacity  of oxygen.

### 4.2 Appearance of a Capacitance Peak after Annealing

The same measurements as those described above were carried out after annealing the electrodes in synthetic air for several hours at the temperature of the subsequent measurement. Poly/dense and epi/dense electrodes were measured after 5, 10, and 15 h of annealing and showed results very similar to those in the pristine state. Figure 5b depicts the capacitance curves of these electrodes after 15 h of annealing with slopes in the low overpotential range (η_WE_ < 100 mV) corresponding to exponential factors α between 0.6 and 0.7. The chemical capacitance curves of the cracked/dense and porous electrodes, however, are completely different already after 6 and 5.5 h at 630 and 460 °C, respectively, compared to the corresponding pristine ones. They exhibit capacitance peaks at about 200 and 150 mV with high maximum values of about 1400 and 2700 F/cm^3^, respectively (Figure 5b). Hence, they show behavior similar to that of (pristine) porous/capped electrodes.

**Figure 7:**
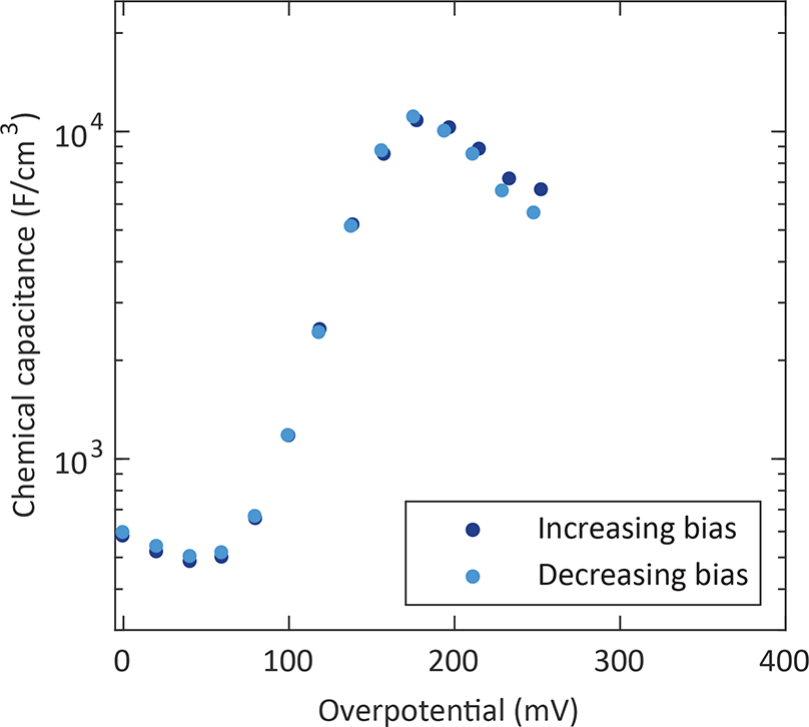
Chemical capacitance of a porous LSC electrode after annealing for about 112 h and after applying bias voltages (*U*_DC_) of up to 440 mV measured at 608 °C.

Figure 7 displays chemical capacitance curves of another porous electrode obtained at 608 °C after an extensive pretreatment, with annealing for about 112 h at temperatures between 460 and 608 °C and bias voltages of up to 440 mV (corresponding to η_WE_ values of up to 385 mV). The chemical capacitance was probed by increasing the bias to an overpotential (η_WE_) of about 390 mV and back to 0 mV. Note that the chemical capacitance was only evaluated up to overpotentials of about 250 mV due to an increase of the intermediate-frequency feature and its merging with the low-frequency semicircle at higher overpotentials (see analysis of the impedance spectra). The curves for increasing and decreasing bias steps are almost identical, and they exhibit extremely high peak values of approximately 11000 F/cm^3^ at an overpotential of about 175 mV. This demonstrates that the electrode is not irreversibly changed by probing the peak. Rather, any microstructural or chemical phenomena leading to the capacitance peak have already taken place during annealing.

We can also conclude that capacitance peaks are only found for electrodes with increased inner surface (cracked/dense, porous, and porous/capped electrodes). However, we still have to understand why the porous/capped films showed the peak already in the pristine state while the other electrodes required an annealing step. Here, a correlation between the appearance of the chemical capacitance peak, degradation of the oxygen exchange resistance, and strontium segregation comes into play. From the literature, it is known that strontium segregates from the bulk to the surface of LSC thin films upon annealing in air, and many studies indicate that this has a negative impact on the kinetics of the oxygen exchange reaction.^33,56−58^ Degradation in terms of slower oxygen exchange kinetics is also found in the present study: For example, the surface exchange resistance of the porous LSC electrode (at η_WE_ = 0 mV) increased by more than 2 orders of magnitude after annealing for 5.5 h at 460 °C (Figures 4b,c and S1). This degradation is accompanied by the evolution of the chemical capacitance peak as explained above. Note that the defect concentration changes due to oxygen exchange at temperatures between 460 and 608 °C are related to equilibration times of a few seconds (see the frequencies of impedance spectra in Figure 4). The occurrence of the chemical capacitance peak of porous and cracked/dense electrodes, however, always required annealing for several hours. Therefore, we do not consider chemical expansion as a primary factor causing the chemical capacitance peaks.

**Figure 8:**
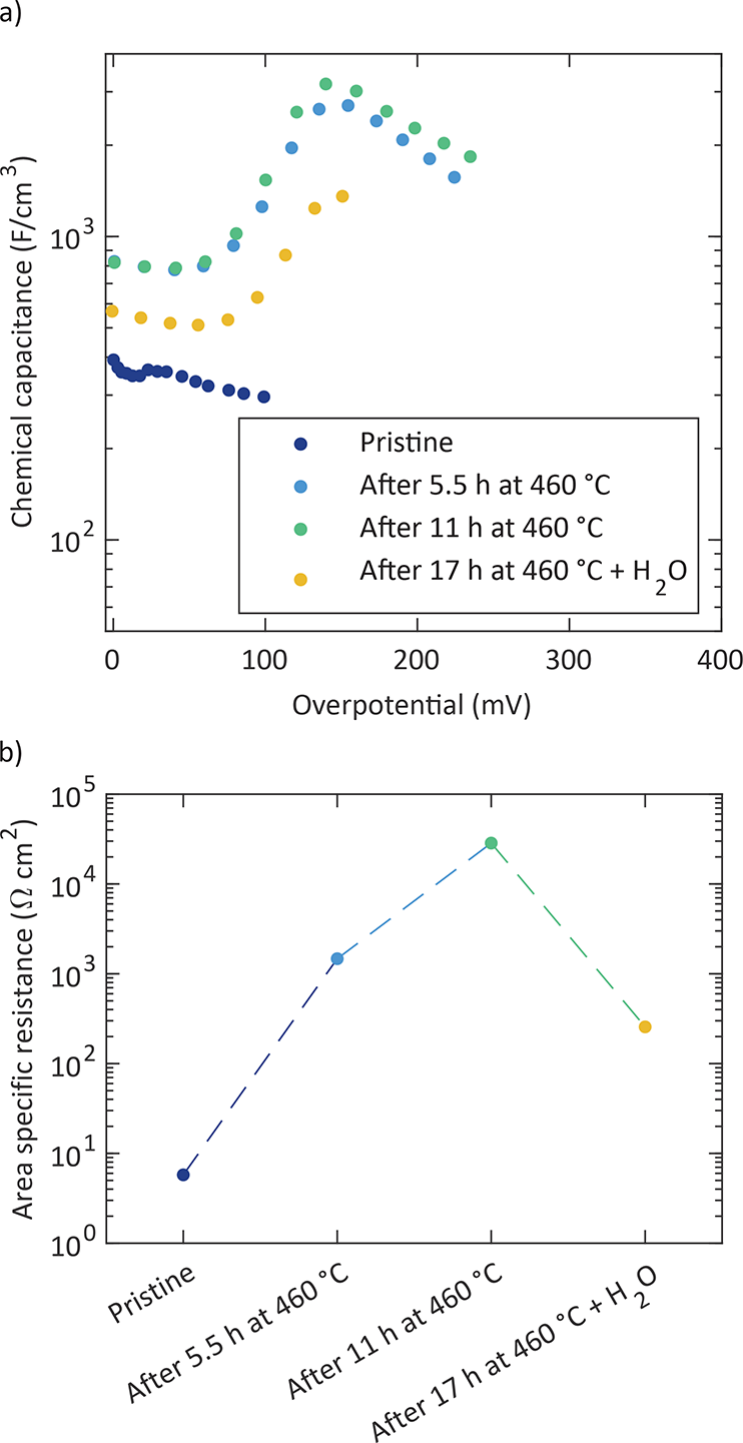
Chemical capacitance of porous LSC electrodes after different pretreatments as a function of the electrode overpotential (a) and the corresponding *R*_s_ at open-circuit conditions (b) measured at 460 °C (lines are a guide to the eye).

Our hypothesis of a relationship between strontium segregation and the appearance of the chemical capacitance peak is also supported by the following experiment (Figure 8).

A porous electrode was measured in the pristine state, after annealing for 5.5 and 11 h at 460 °C, respectively, and after annealing for 17 h and subsequent stirring of the sample in double-distilled H_2_O for 30 min. The electrodes with a thermal history of 5.5 and 11 h exhibit very similar chemical capacitance values and a pronounced peak. However, after the H_2_O treatment, the peak value decreased to less than half compared to the measurement after 11 h at 460 °C. Figure 8b shows the corresponding surface exchange resistances at open-circuit conditions. The two annealing steps increased the resistance by nearly 4 orders of magnitude, which we assume to be related to strontium segregation. The H_2_O treatment clearly lowers the resistance in accordance with former studies^33,59^ reporting enhanced oxygen exchange kinetics after removal of a surface strontium species by H_2_O. Hence, the lower surface exchange resistance found here after the H_2_O treatment suggests at least partial removal of a water-soluble surface strontium species. A more detailed analysis of the relationship between strontium segregation and the capacitance peak is presented below.

### 4.3 Appearance of a Capacitance Peak after High Bias Treatment

**Figure 9:**
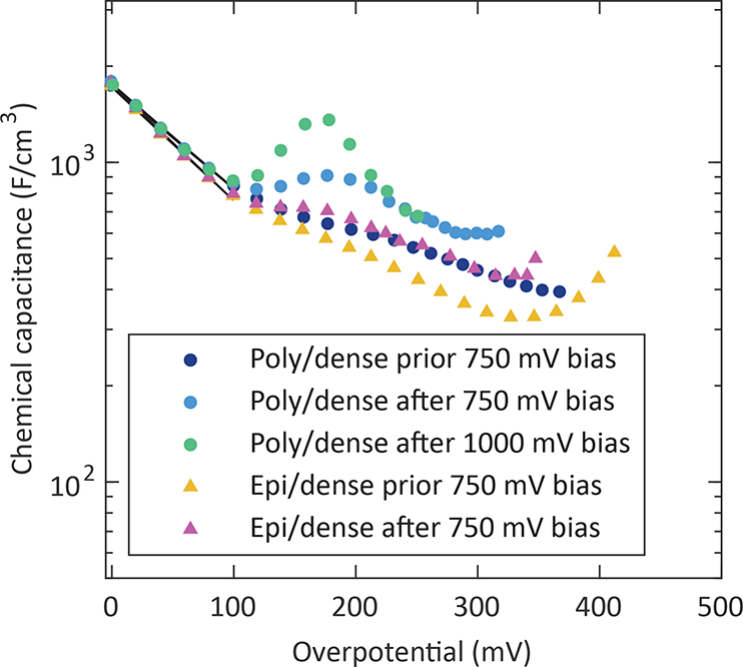
Chemical capacitance of annealed poly/dense and epi/dense electrodes (measured at 608 °C) before and after a high anodic bias voltage (*U*_DC_) was applied for 1 h and their corresponding fits (solid line).

Apart from thermal pretreatments, we also investigated the chemical capacitance after applying high anodic bias voltages. Figure 9 shows chemical capacitance curves measured under moderate overpotentials after a high anodic bias voltage *U*_DC_ of 750 or 1000 mV was applied for 1 h to poly/dense and epi/dense microelectrodes.

It should be noted that those films did not show a chemical capacitance peak after annealing (Figure 5b). Even a thermal history of more than 500 h at 608 °C did not lead to a chemical capacitance peak for a poly/dense electrode (Figure S2). Interestingly, anodic polarization for 1 h at *U*_DC_ = 750 mV (η_WE_ = 406 mV) led to a small capacitance peak for poly/dense films. Also epi/dense films showed a small indication of a peak after such a bias treatment. Moreover, applying *U*_DC_ = 1000 mV (η_WE_ = 438 mV) for 1 h to a poly/dense electrode caused a substantial chemical capacitance peak of about 1400 F/cm^3^. In this case, inspection with an optical microscope revealed that the corresponding electrode had undergone morphological changes due to this harsh bias treatment. A lower bias voltage of *U*_DC_ = 200 mV applied for more than 500 h, however, did not yield a significant *C*_chem_ increase (Figure S3). Accordingly, poly/dense films require the application of high bias voltages for developing a chemical capacitance peak. It is noteworthy that, at low overpotentials (<100 mV), the chemical capacitance of all poly/dense as well as epi/dense electrodes did not change at all (α = 0.6).

**Figure 10:**
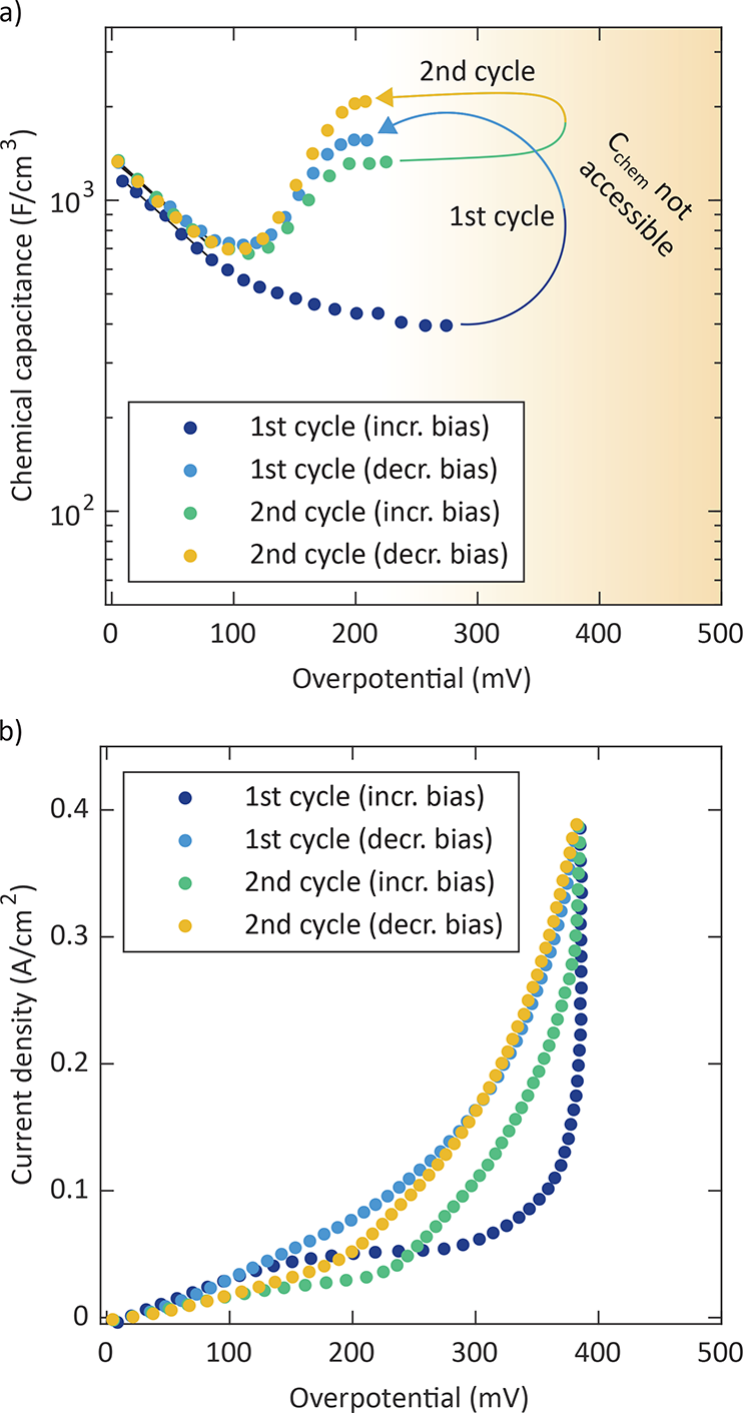
(a) Chemical capacitance of a cracked/dense electrode at a temperature of 600 °C that was cycled twice up to *U*_DC_ = 1000 mV and back to 0 mV (with 20 mV steps). For high bias voltages, the chemical capacitance was not accessible from analysis of the spectra (indicated by the marked area). (b) Corresponding current density.

In a further measurement series, we cycled different microelectrodes stepwise up to high bias voltages (*U*_DC_ = 1000 mV; η_WE_ = 384 mV) and back to 0 mV. Because of the long measurement time (44 h), this inherently combined annealing and voltage treatment. Figure 10a shows the resulting curves of a cracked/dense electrode for two such cycles.

Each cycle consisted of a forward run (increasing bias) and a subsequent reverse run (decreasing bias). We again see that at first the chemical capacitance decreases with increasing overpotential, as expected for a decreasing oxygen vacancy concentration. However, already in the first reverse run a very pronounced capacitance peak becomes visible. Note that for overpotentials higher than 275 mV the spectra cannot be analyzed properly, and thus chemical capacitances are not extractable there. During the second cycle, the peak remains with only small changes in the forward run and an increased peak value in the reverse run. This further demonstrates the reproducibility of the chemical capacitance peak and that it is caused by a process occurring under high polarization at the end of the first forward run, i.e., at higher overpotentials than the peak detection itself. The corresponding current of the two cycles is given in Figure 10b. The strong increase in the first forward run at the highest voltages indicates some drastic changes of the surface reaction kinetics, which seems to be associated with some permanent morphological changes of the electrode because the currents of all of the following cycles increased at overpotentials higher than 250 mV in comparison to the first run. These bias-induced changes differ from those triggered by the annealing itself because the polarization resistance was decreased here.

**Figure 11:**
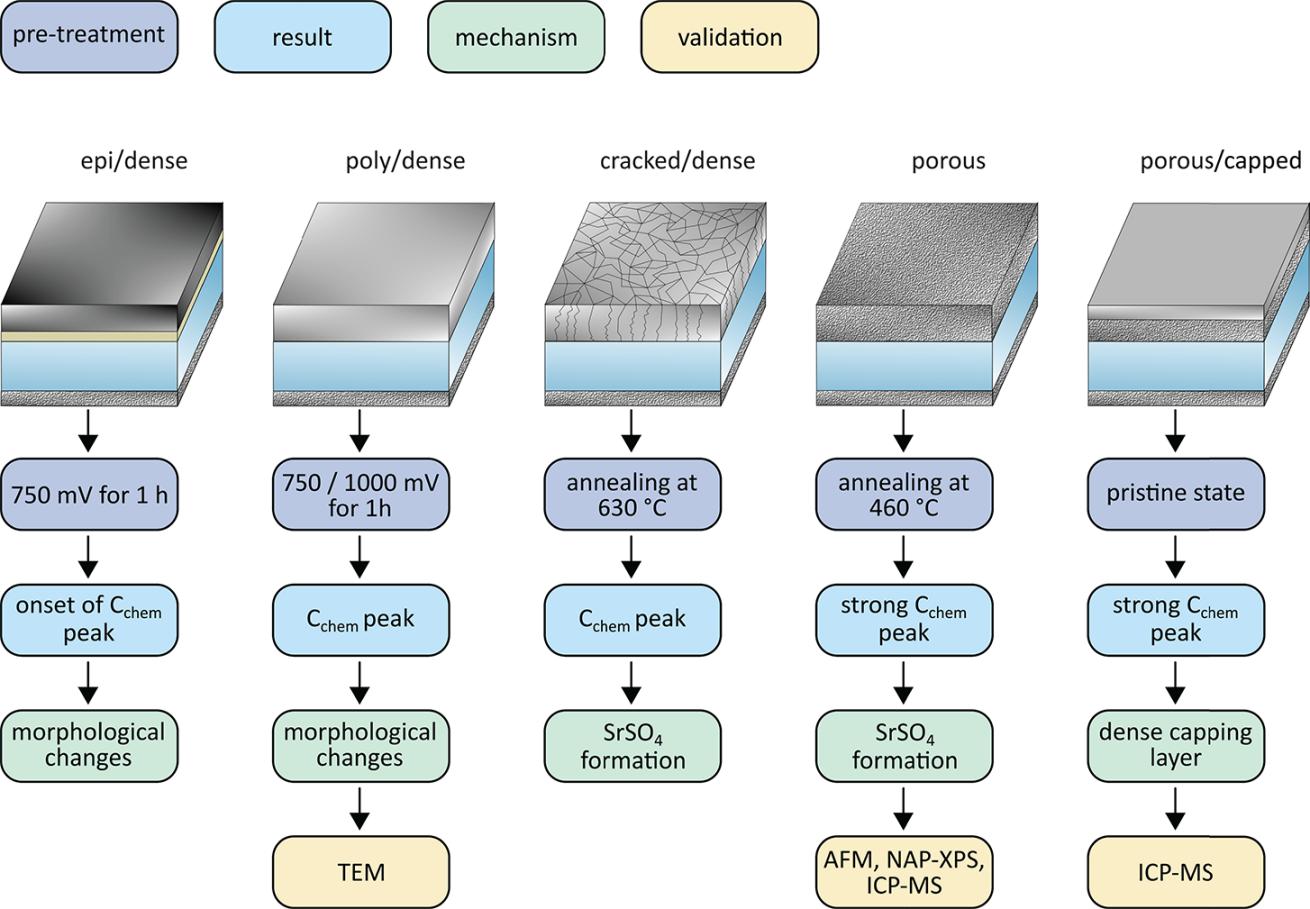
Schematic of the different samples, pretreatments, results, corresponding underlying mechanisms, and analytical techniques employed for their justification.

The sketch in Figure 11 summarizes the different samples, pretreatments, results, corresponding underlying mechanisms, and employed experiments justifying this interpretation. The suggested mechanisms and their validation are elucidated in detail in the subsequent discussion.

## 5 Mechanistic Model and Its Validation

### 5.1 Degradation Mechanisms Causing Chemical Capacitance Peaks

We now introduce a mechanistic model explaining the described chemical capacitance peaks and their dependence on the samples’ pretreatment and microstructure. Regardless of the electrode type or pretreatment, all capacitance peaks are similarly shaped and are found at similar overpotentials. Therefore, we suggest that they all can be traced back to the same underlying mechanism. As shown in a former study^29^ and briefly discussed above, porous/capped electrodes exhibit a capacitance peak already in the pristine state due to high pressure oxygen gas formation in closed pores. Closed porosity seems to be the key requirement for the occurrence of the capacitance peak because this is the distinctive property of pristine porous/capped electrodes. Thus, we conclude that all other pristine films do not show a capacitance peak due to the absence of closed pores and that either annealing or treatment with high bias voltages induces such closed pores and the possibility of filling them with highly pressurized oxygen under anodic polarization. This then manifests itself in chemical capacitance peaks.

**Figure 12:**
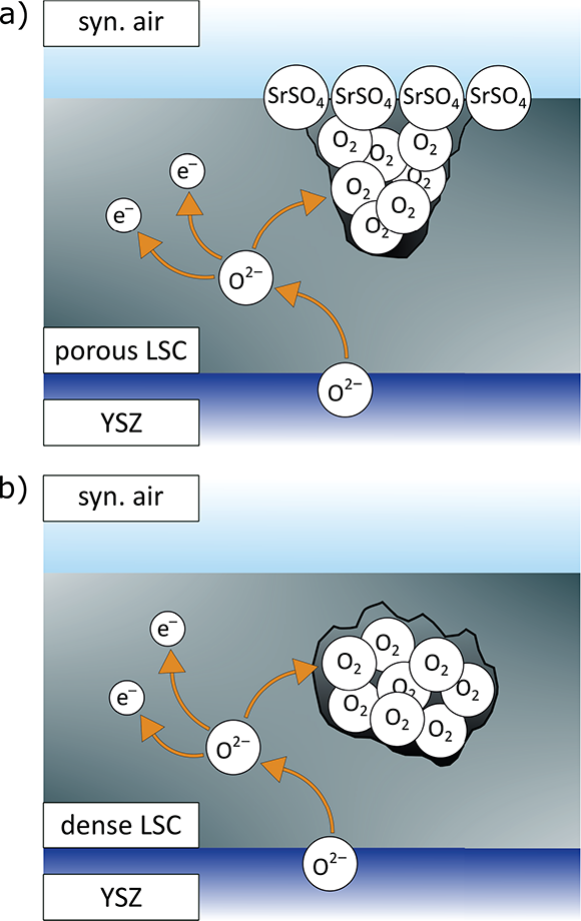
Sketch of the formation of high-pressure oxygen in pores of a porous film that get closed due to a SrSO_4_ phase formed upon annealing (a) and in closed pores of a dense film caused by high bias treatment (b).

Despite the same origin of the capacitance peaks in all films (pressurized oxygen in closed pores), the mechanism of forming those closed pores may be manifold. In our case, we seem to face two different mechanisms. The first refers to electrodes with open pores or cracks in the pristine state (porous and cracked/dense). Here we propose that upon annealing in synthetic air, strontium segregates from the bulk of the LSC film to the surface, as revealed in former studies.^33,56,57^ Initially, this surface strontium may exist in the form of SrO or a SrO termination layer.^33,57,60^ However, LSC is known to be very prone to sulfur poisoning from the gas phase,^19,20^ and in accordance with the literature,^61,62^ we expect that, even for annealing in synthetic air, minute traces of sulfur cause the formation of SrSO_4_ particles. We suppose that those particles grow at crack or pore surfaces and finally reach a size that leads to the closure of open pores or cracks (see the sketch in Figure 12a). This process leads to higher surface exchange resistances as well as to the appearance of a capacitance peak, as depicted in Figure 8 for a porous electrode. A further validation of this hypothesis by means of surface-sensitive analytical techniques is given in the subsequent section.

Because of the absence of open pores or cracks, this mechanism does not work for dense electrodes (poly/dense and epi/dense), despite strontium segregation. There, we suppose that high anodic bias voltages lead locally to such a high mechanical load that morphological changes take place, ultimately resulting in closed pores or cracks in dense films, as sketched in Figure 12b. As described above, for a bias voltage of 1000 mV, such morphological changes were already visible in the optical microscope. The formation of such closed pores after a bias treatment was also confirmed via cross-sectional TEM measurements (see below).

### 5.2 Analysis of Surface Species

#### 5.2.1 ICP-MS

For electrodes with open inner surfaces (porous and cracked/dense), we suggested that strontium segregation during annealing plays an important role for the closure of open pores or cracks. For the purpose of validation, we analyzed the surface compositions of pristine and annealed LSC thin films of different sample types via ICP-MS measurements using an approach already applied in previous studies.^29,30,33,59^ Furthermore, by employing this method, we can get information about the morphology of the studied LSC films, which is another important aspect of our mechanistic model.

As shown in the literature,^30,33,59^ a water-soluble strontium species may form on the surface of LSC films. The amount of this species and a possible dependence on the film microstructure and annealing treatments were examined by stirring the films in pure H_2_O (particularly leaching surface species) and then dissolving them in HCl, in both cases followed by a subsequent chemical analysis of the solute via ICP-MS. Two equally prepared samples were investigated: one was analyzed in its pristine state, and the other one was annealed for 15 h at 608 °C in synthetic air prior to the ICP-MS measurements. After the treatment with pure H_2_O and the first ICP-MS measurement, the films were completely dissolved in HCl to determine the amount of strontium in the bulk (*c*_bulk_, including surface species that are not soluble in H_2_O). The amount of (water-soluble) surface strontium (*c*_surf_) was related to the amount of strontium in the entire film (*c*_total_ = *c*_surf_ + *c*_bulk_). Thereby, it was possible to compare the results of different sample types. Table 2 displays the corresponding results for poly/dense, porous, and porous capped films.

**Table 2:** Ratio of Water-Soluble Surface Strontium (*c*_surf_) to Total Strontium (*c*_total_ = *c*_surf_ + *c*_bulk_) of LSC Thin Films

sample type	*c*_surf_/*c*_total_ (%) of the pristine state^29^	*c*_surf_/*c*_total_ (%) after 15 h at 608 °C
poly/dense	0.86	2.00
porous	6.75	5.86
porous/capped	1.09	3.99

Assuming a homogeneous distribution of the water-soluble strontium species across the entire surface, these measurements give information on the water-accessible surface area and thus also on the morphology of the respective film. Analysis of the pristine films was already part of an earlier study,^29^ which identified similar amounts of water-soluble surface strontium for poly/dense and porous/capped films. Accordingly, the top layer of the porous/capped electrode has a morphology similar to that of the poly/dense electrode and thus indeed closes the open pores in the bottom layer. This is the reason for the appearance of the capacitance peak already in the pristine state of porous/capped films and also for the absence of any further changes after annealing (Figure 6) because strontium surface species formed upon annealing did not contribute to a substantial increase of the closed porosity. Porous films, on the other hand, have large amounts of water-soluble surface strontium (Table 2), indicating open porosity with a 7–8 times larger accessible surface area than poly/dense films, in agreement with an earlier study.^30^

After annealing, the amount of water-soluble surface strontium strongly increased in the case of poly/dense and porous/capped films, as expected due to strontium segregation to the surface.^33,56,57^ However, the porous film seems to have less strontium on the surface after the annealing process compared to its pristine state. This supports our suggested model because we suppose that, during annealing in synthetic air, a less-soluble strontium-containing species is formed (SrSO_4_) that causes the closure of part of the open pores. Hence, the water-accessible surface area is reduced, leading to a slight decrease of water-soluble strontium species, despite further strontium segregation during the annealing process. Moreover, this is also in agreement with the fact that the H_2_O treatment only lowers the capacitance peak (Figure 8a), i.e., only partially reopens the pores.

These results reveal that strontium indeed segregates from the bulk to the surface of our LSC films, thus supporting the described degradation mechanism. The strontium-rich surface phase formed upon annealing was investigated in more detail by NAP-XPS (see the next section).

#### 5.2.2 *In Situ* NAP-XPS

*In situ* NAP-XPS was used to further investigate the surface chemistry of LSC thin films. Impedance measurements were conducted with porous and poly/dense rectangular microelectrodes while recording XPS spectra at 1 mbar of oxygen pressure. Anodic bias was applied, and the corresponding chemical capacitance was analyzed as described above. Hence, in addition to analyzing the composition of the surface species, we could investigate whether these species change under conditions where the chemical capacitance peak is found.

The lower oxygen pressure of 1 mbar inside the XPS chamber compared to the *ex situ* measurements had to be considered for this analysis. According to Nernst’s equation, an additional 84 mV is necessary at 460 °C to yield the same oxygen chemical potential as that in air. An *ex situ* measurement in air as well as at 1 mbar of oxygen partial pressure confirmed this consideration: The onset of the corresponding capacitance peak indeed shifted by almost 80 mV (Figure S5a). This shows, in accordance with former studies,^40,41^ that the chemical capacitance solely depends on the chemical potential of oxygen in the working electrode (eq 7).

In order to observe any bias-induced chemical changes at the surface, we recorded O 1s spectra (Figure S5b). In general, all obtained O 1s spectra consist of two distinctive signals, which were fitted according to three components. The component at high binding energy (531.5 eV) is usually identified as the surface component (O 1s surf), whereas the component at low binding energy (528.5 eV) is generally considered as “bulk” oxygen.^36,63−65^ The O 1s bulk peak is strongly asymmetric, and only the addition of a third species close to the main bulk peak leads to a well-converging fit. This asymmetry is attributed to the metal-like electronic structure of LSC.^45,46^ More details on this asymmetric feature can be found in the Supporting Information.

O 1s spectra were recorded on a porous electrode, which was annealed for more than 25 h at 460 °C in synthetic air prior to the NAP-XPS measurement. *In situ* impedance spectra revealed a capacitance peak at an overpotential of about 243 mV at 460 °C, which is in accordance with the expected position when taking account of the lower oxygen pressure of 1 mbar inside the XPS chamber. Figure S5b displays the corresponding O 1s signals from simultaneously performed XPS measurements. Neither a significant peak shift nor a strong change of the intensities was found for the XPS spectra at 243 mV compared to the spectra at open-circuit conditions.

In addition, O 1s spectra were also recorded on a poly/dense electrode, pretreated with a bias voltage of *U*_DC_ = 750 mV for 1 h. As shown above, such electrodes exhibited a chemical capacitance peak after the application of this high bias voltage. Again, because of the lower oxygen pressure of 1 mbar, the capacitance peak was observed at 245 mV. As in the case of the annealed porous electrode, O 1s spectra recorded at the capacitance maximum-related overpotential and at open-circuit conditions are very similar (Figure S5b). Thus, there seems to be no XPS-accessible surface redox process that can be directly related to the chemical capacitance peak at the respective anodic overpotential. Rather, for both porous and dense electrodes, the processes relevant for the existence of a capacitance peak have already taken place during the different pretreatments, i.e., annealing and application of a high anodic bias voltage. This is in accordance with our mechanistic model, suggesting a closed porosity as the main cause for the capacitive peak.

**Figure 13:**
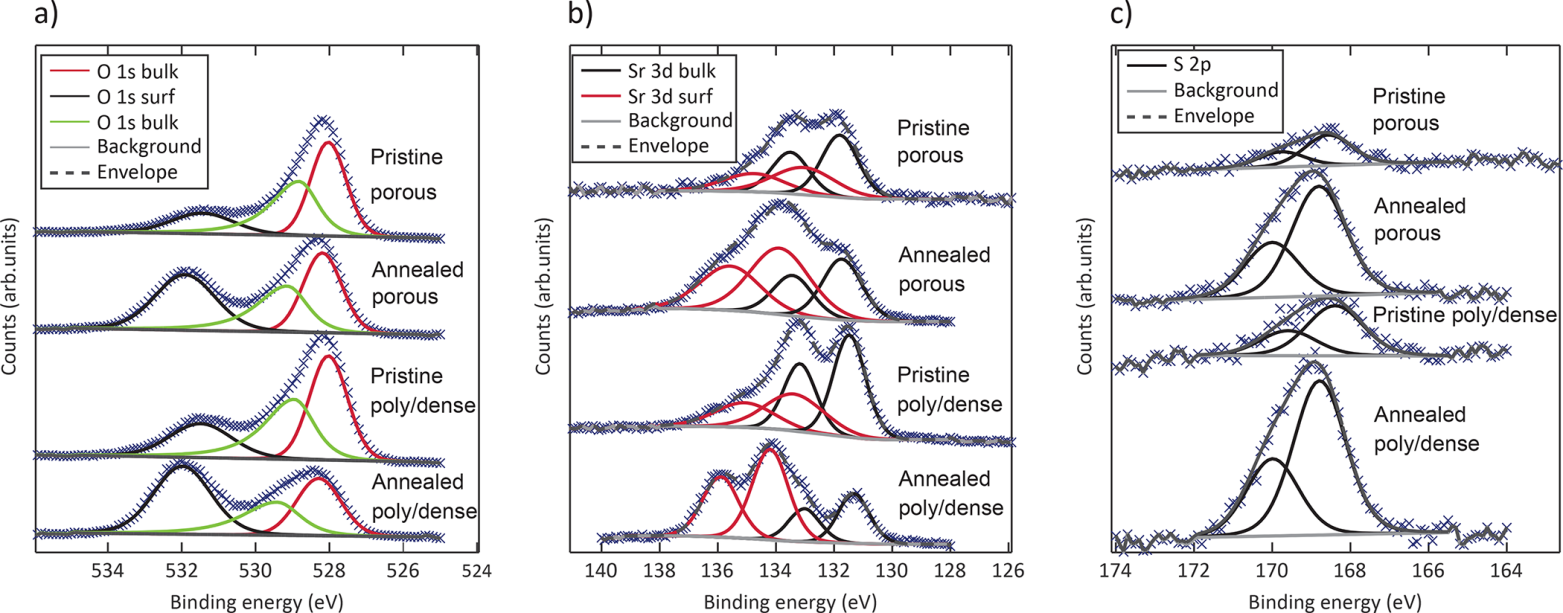
O 1s (a), Sr 3d (b), and S 2p (c) spectra of pristine and annealed poly/dense and porous electrodes (counts marked by blue crosses).

**Figure 14:**
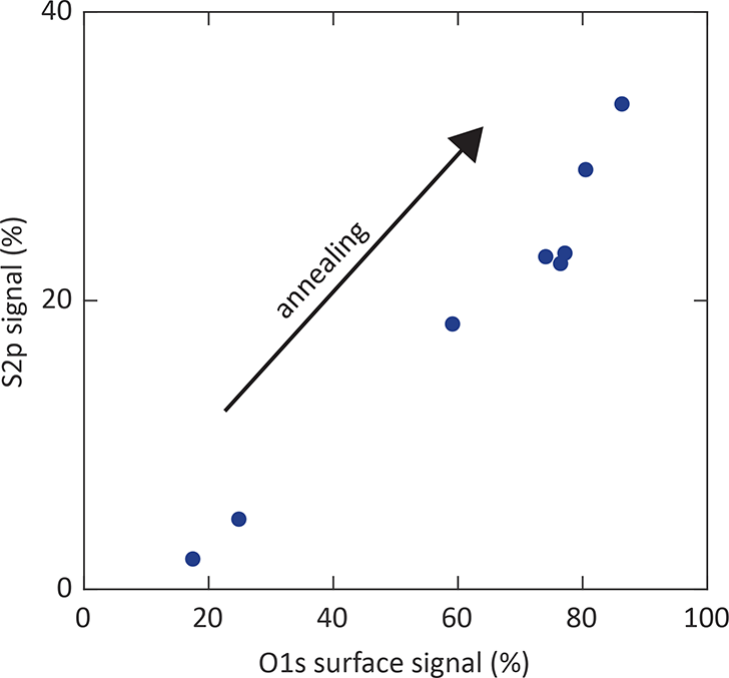
S 2p signal plotted against the O 1s surface signal (both signals are related to the total cation counts).

For analysis of the thermally induced degradation in more detail, O 1s, Sr 3d, and S 2p spectra of pristine and annealed (*ex situ* in synthetic air) poly/dense and porous electrodes were recorded under open-circuit conditions (Figure 13). The intensities of the surface-related O 1s and Sr 3d (Sr 3d surf) signals and particularly the S 2p-related species increased after annealing in synthetic air. Corresponding *in situ* impedance measurements revealed an increase of *R*_s_ by several orders of magnitude after annealing. Additional *in situ* measurements with a cathodic overpotential of about 230 mV neither changed the capacitance peak of a subsequent anodic measurement nor had an effect on the simultaneously recorded S 2p signal. The total S 2p signal is plotted against the surface O 1s signal after various annealing times (Figure 14), yielding a linear correlation between these signals. This indicates that the surface O 1s signal is mainly caused by a sulfur-containing species. In combination with the Sr 3d signal, it can be concluded that the phase on the surface mainly consists of sulfur, strontium, and oxygen. Accordingly, despite the use of very clean gases (i.e., 99.999% purity) in all experiments, formation of SrSO_4_ occurs during long annealing times, in agreement with the results of previous studies.^20,61,62,66,67^ This probably also caused severe degradation of the oxygen exchange kinetics of the LSC thin films measured here (Figure S1). Moreover, we may conclude that the large grains visible in the AFM scan of the annealed porous film (Figure 3) consist of this SrSO_4_ phase. This is in line with recent studies,^66,68^ which showed that trace amounts of sulfur (ca. 0.5 ppmv) are present in typical measurement setups even when using high-purity measurement gases. Also SrCO_3_ may form due to trace amounts of CO_2_. However, carbonates are supposed to desorb at the temperatures used in this study.^69^

Hence, these findings are in excellent agreement with the suggested mechanism that initially open pores or cracks become closed during annealing as a result of SrSO_4_ formation. Under anodic polarization, high-pressure oxygen then forms in these closed pores, leading to the observed chemical capacitance peak. Remarkably, these closed pores seem to withstand pressures in the range of 10^4^ bar (calculated via the Soave–Redlich–Kwong real-gas equation, as shown in a previous study^29^) because consecutive measurements of the chemical capacitance on a porous electrode yielded almost identical curves (Figure 7).

### 5.3 Identification of Voltage-Induced Morphological Changes

**Figure 15:**
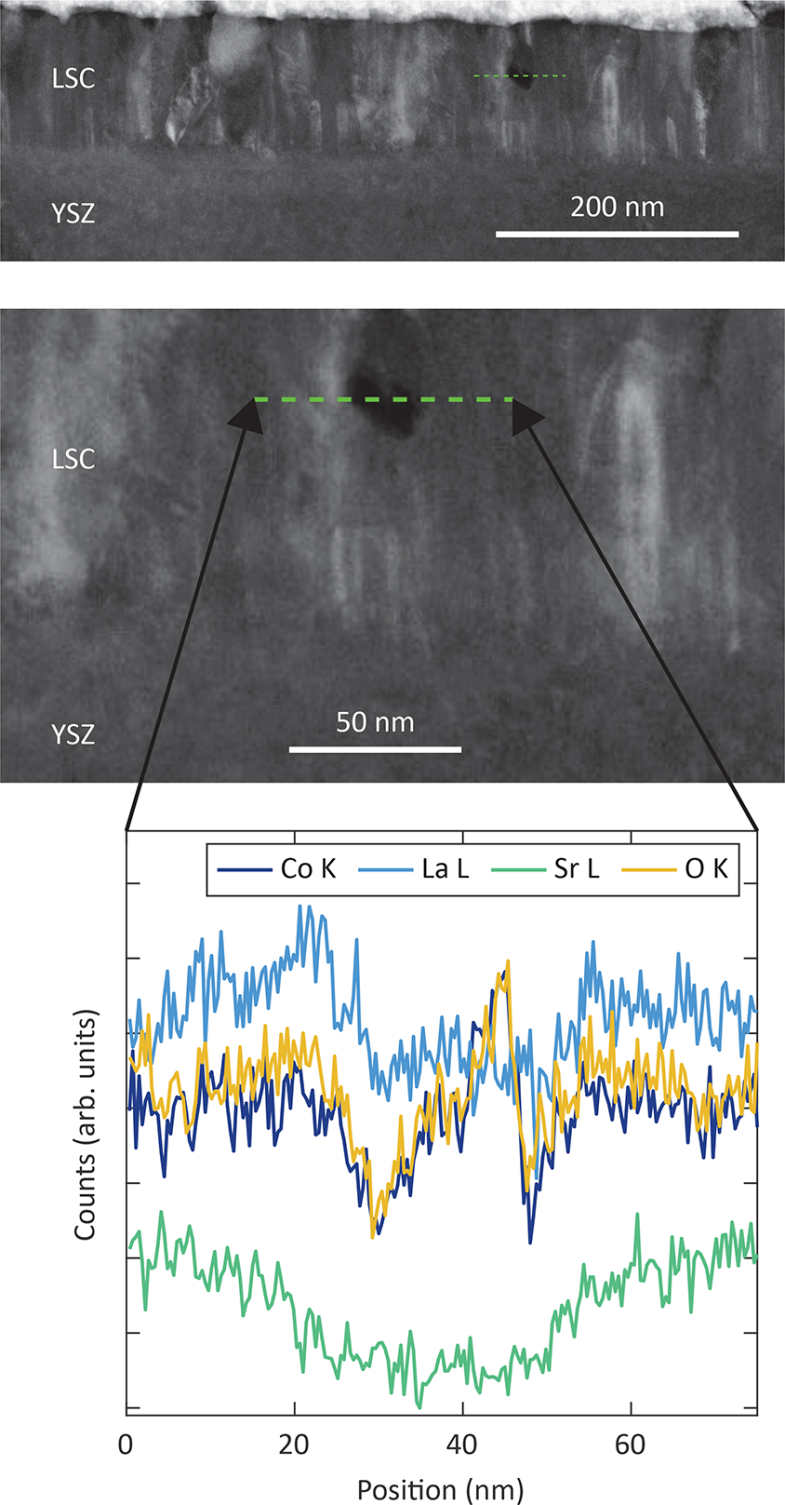
HAADF-STEM measurement (top image and section thereof in the middle) and EDX analysis (bottom) of the green marked area of a poly/dense electrode after 750 mV was applied for 1 h.

**Figure 16:**
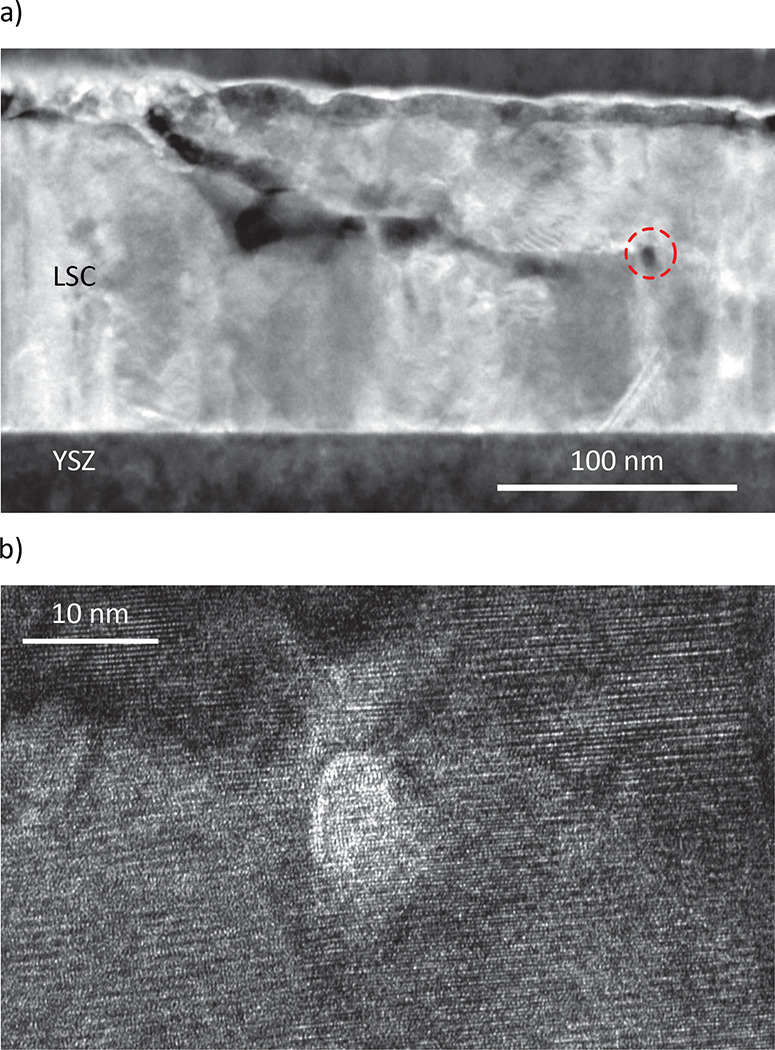
(a) HAADF-STEM measurement of a poly/dense electrode after 750 mV was applied for 1 h, revealing a closed pore (marked with dashed red line) and a crack in the bulk of the film. (b) BF-TEM image of the closed pore shown in part a.

TEM and HAADF-STEM measurements were performed on a lamella with 10 μm length, prepared from a poly/dense electrode after a bias voltage of *U*_*DC*_ = 750 mV was applied for 1 h at 608 °C. The chemical capacitance analysis of this film is displayed in Figure 9, revealing a peak at an overpotential of approximately 175 mV. According to our model, this peak indicates bias-induced formation of closed pores. HAADF-STEM measurements and EDX scans confirmed the existence of such closed pores because the elemental counts of the respective areas are significantly lower, as shown in Figure 15. At the position of about 45 nm in the EDX scan, an increase of both the cobalt and oxygen signals is obtained. This may be associated with the small protrusion in the area of the investigated closed pore. The corresponding increase of the cobalt and oxygen signals could result from a Co_3_O_4_ phase, which may have formed under anodic polarization, as was similarly found in a former study^70^ for an LSCF oxygen electrode after SOEC operation. A BF-TEM image of the closed pore in Figure 15 is displayed in Figure S6, where the brighter area indicates the position of the pore (marked with a dashed red line). Another closed pore in the bulk of this poly/dense film is shown in the HAADF-STEM image of Figure 16a. The corresponding BF-TEM image of this closed pore is depicted in Figure 16b. On the left next to this closed pore, a crack seems to extend from the bulk to the surface or a near-surface layer (Figure 16a).

In line with our suggested model mechanism, anodic bias voltages of ≥750 mV (i.e., electrode overpotentials of >400 mV) may thus lead to mechanical failure of our dense films and the formation of closed pores in the respective electrodes. Unlike other studies on LSCF electrodes,^13,16,22,26^ which reported pore and crack formation at the electrode/electrolyte interface and delamination of the electrode from the electrolyte or the barrier layer, here it seems that pores and cracks form in the bulk of the electrode. This may indicate a very good adhesion of our poly/dense LSC electrodes to the YSZ electrolyte.

### 5.4 Porosity Estimation Based on a Real-Gas Model

From all of these results, we conclude that closed porosity is necessary for obtaining the observed chemical capacitance peak under anodic polarization. These closed pores result either from deposition of a dense capping layer on top of a porous electrode (porous/capped) or from two different degradation phenomena: (i) Upon annealing cracked/dense or porous electrodes for several hours in synthetic air, minute traces of sulfur-containing species react with a SrO phase formed due to strontium segregation to the surface and probably also pull out further strontium from the film to form large SrSO_4_ particles. This SrSO_4_ causes the closure of at least a part of the initially open pores (Figure 12a). (ii) The second degradation mechanism involves mechanical failure due to the application of high bias voltage (*U*_DC_ ≥ 750 mV). HAADF STEM and EDX measurements revealed closed pores in the bulk of poly/dense films after such bias treatments (Figure 12b). For both degradation processes, subsequently recorded impedance spectra revealed chemical capacitance peaks between 140 and 200 mV (in synthetic air).

The same kind of capacitance peaks also result for porous/capped electrodes, where a closed porosity was intentionally introduced during the fabrication process. In ref (29), a detailed model was introduced to quantify these capacitance peaks by considering the formation of highly pressurized oxygen in closed pores using a real-gas equation. By including pressure values  and fugacity coefficients ϕ determined from the Soave–Redlich–Kwong real-gas equation, the chemical capacitance can be calculated as follows:
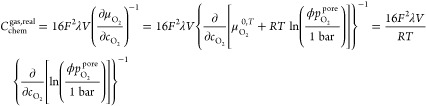
12with the film porosity λ and μ_O_2__ (=2 μ_O_) and *c*_O_2__ (=*c*_O_/2) being the chemical potential and concentration of O_2_, respectively. By optimization of λ with a least-squares method, it is possible to estimate the volume fraction of closed porosity contributing to a measured capacitance peak.

**Figure 17:**
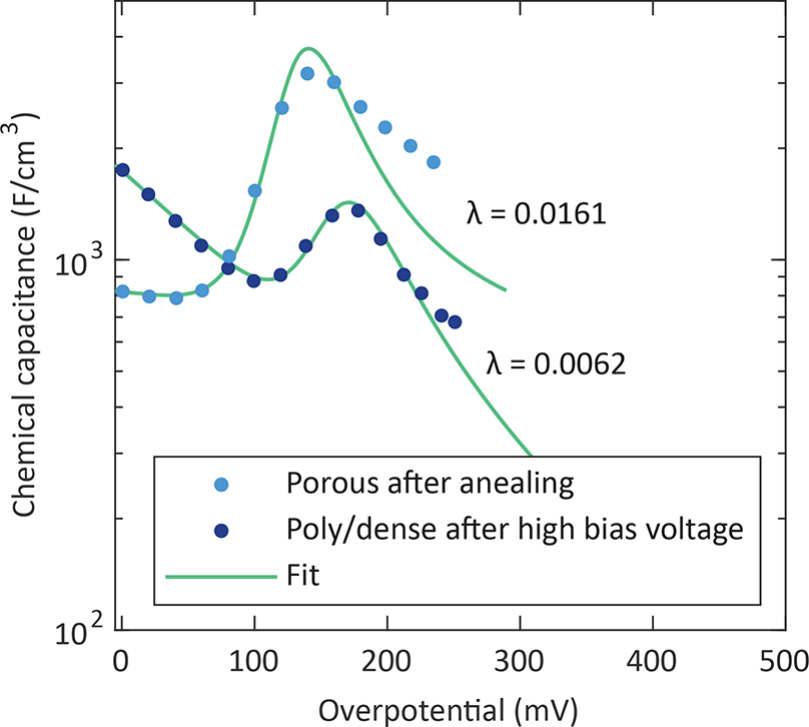
Chemical capacitance curves of a porous electrode after annealing for 11 h at 460 °C and a poly/dense electrode after *U*_DC_ = 1000 mV was applied for 1 h at 608 °C. The green solid line represents the sum of the extrapolation of the capacitance at low overpotentials and the calculated capacitance according to eq 12. Closed porosity values (λ) from the optimization are given for both electrodes.

This was done for both of the above-described degradation cases, i.e., for an annealed porous electrode and for a poly/dense electrode after high anodic bias voltage was applied. Figure 17 displays the experimentally obtained capacitances and the fit results for these two cases. The green solid fit lines represent the sum of (i) the extrapolation of the defect-related chemical capacitance at low overpotentials and (ii) the numerically determined capacitance according to eq 12 with pressure and fugacity coefficient values from the Soave–Redlich–Kwong real-gas equation.^29,71^ A more detailed description of this model calculation and the associated real-gas equation is provided in the Supporting Information.

Both calculated curves predict the capacitance increase and the shift of the capacitance peak to higher overpotentials with increasing measurement temperature extremely well, which supports our suggestion of high pressure oxygen formation and storage in closed pores being the responsible mechanism. Deviations at high overpotentials for the porous electrode may be ascribed to some errors in determining the overpotential of the porous electrode at higher bias voltages due to an increase of intermediate-frequency features (see above) or to some leaks in the closed pores, which lowers the true fugacity.

On the basis of the employed real-gas equation, we find pressure values of up to about 10^4^ bar. Obviously, closed pores seem to endure enormous mechanical gas pressures. Even pores that were closed by a SrSO_4_ phase can apparently withstand such high gas pressures because the capacitance peaks of an annealed porous electrode were almost identical when cycling up to an overpotential of 385 mV and subsequently down to 0 mV (Figure 7). Hence, the capping SrSO_4_ phase seems to have high mechanical stability at the surface of LSC films. The model calculation yields porosity values of λ = 0.0161 for the porous electrode and λ = 0.0062 for the poly/dense electrode. Accordingly, such electrochemical measurements may be used as an online nondestructive observation tool to detect the formation of closed pores caused by degradation phenomena at an early stage and allows determination of a closed porosity with high sensitivity. A detection limit of λ ≈ 5 × 10^–4^ for a porous electrode and λ ≈ 2 × 10^–3^ for a dense electrode can be estimated based on the data in Figure 17. Note that the detection limits vary between the sample types due to the different slopes at low overpotentials; i.e., for steeper slopes, higher porosity values are required to identify peaks in the chemical capacitance curve.

## 6 Conclusion

Impedance spectroscopy was used to analyze the chemical capacitance of LSC thin-film microelectrodes with different microstructures under varying anodic bias voltages. The pristine films exhibit a decrease of the chemical capacitance with increasing anodic overpotential, as expected from the decrease of the oxygen vacancy concentration in this regime. However, different types of pretreatments cause severe changes from this behavior, with an increase of the chemical capacitance under anodic overpotentials and a very pronounced capacitance peak at 150 mV and 460 °C in air. Different oxygen partial pressures and temperatures shift the peak positions in accordance with Nernst’s equation. The first type of pretreatment causing a capacitance peak simply consists of annealing electrodes with open inner surfaces (pores or cracks) for a few hours between 460 and 630 °C in synthetic air. After such an annealing step, these electrodes exhibit a capacitance peak that hardly changes by its monitoring under bias itself. The second type of pretreatment involves the application of a high anodic bias corresponding to electrode overpotentials of >400 mV. Following such a bias treatment, even polycrystalline dense electrodes without open inner surfaces show a peak of the chemical capacitance. These peaks are very similar to those found for porous electrodes, which were intentionally capped with a dense layer already during the fabrication process. There, high-pressure oxygen gas formed in closed pores and high fugacity coefficients of the corresponding real gas are the reasons for the chemical capacitance peak.

The formation of closed pores is also the reason behind the capacitance peaks found in annealed and bias-treated films. ICP-MS, AFM, and *in situ* NAP-XPS measurements suggest that annealing in synthetic air leads to the closure of already existing open pores or cracks due to strontium segregation and the formation of a SrSO_4_ phase on the surface of the respective films. Furthermore, TEM and EDX measurements revealed the formation of closed pores in dense electrodes as a result of bias-induced morphological changes in the bulk of these films. Model calculations based on a real-gas equation agree well with experimental data, implying that pressures of up to 10^4^ bar may develop in closed pores formed due to the described degradation phenomena. Moreover, such model calculations allow one to determine the amount of closed porosity in the measured films (in the range of 1% in our case). An even much lower detection limit can be estimated, and thus such capacitance measurements may also be employed as a nondestructive online measurement tool to identify possibly destructive loads in SOEC systems at an early stage.

## Supporting Information Available

Surface exchange resistance of pristine and annealed electrodes of all different sample types (Figure S1), additional chemical capacitance results of poly/dense electrodes during and after annealing for more than 500 h at 608 °C (Figures S2 and S3), setup of *in situ* NAP-XPS (Figure S4), chemical capacitance of an annealed porous electrode in synthetic air and at 1 mbar of oxygen partial pressure (Figure S5a), O 1s spectra of annealed poly/dense and porous electrodes at open-circuit conditions and at about 245 mV overpotential (Figure S5b), additional BF-TEM image of a poly/dense electrode after 750 mV was applied for 1 h (Figure S6), and model calculation and real-gas model for porosity estimation (Figure S7) (PDF)
