# Supplementary material for: Closed-Pore Formation in Oxygen Electrodes for Solid Oxide Electrolysis Cells Investigated by Impedance Spectroscopy
Source: ACS Appl Mater Interfaces. 2023 Feb 2;15(6):8076–92. doi: 10.1021/acsami.2c20731 (PMC9940111; doi:10.1021/acsami.2c20731)
Supplement: Supplementary file 1 — am2c20731_si_001.pdf [file am2c20731_si_001.pdf]

Supporting Information: Closed Pore Formation in Oxygen Electrodes for Solid Oxide Electrolysis Cells Investigated by Impedance Spectroscopy

Martin Krammer,[*,†] Alexander Schmid,[†] Andreas Nenning,[†] Andreas Ewald Bumberger,[†] Matthäus Siebenhofer,[†,‡] Christopher Herzig,[†] Andreas Limbeck,[†] Christoph Rameshan,[¶,§] Markus Kubicek,[†] and Juergen Fleig[†]

†*Institute of Chemical Technologies and Analytics, TU Wien, Getreidemarkt 9/164-EC, 1060 Vienna, Austria*

‡*Centre for Electrochemical Surface Technology GmbH, Viktor-Kaplan-Straße 2, 2700 Wiener Neustadt, Austria*

¶*Institute of Material Chemistry, TU Wien, Getreidemarkt 9/165-PC, 1060 Vienna, Austria.*

§*Chair of Physical Chemistry, Montanuniversität Leoben, Franz-Josef-Straße 18, 8700 Leoben, Austria.*

E-mail: martin.krammer@tuwien.ac.at

1 Surface exchange resistance

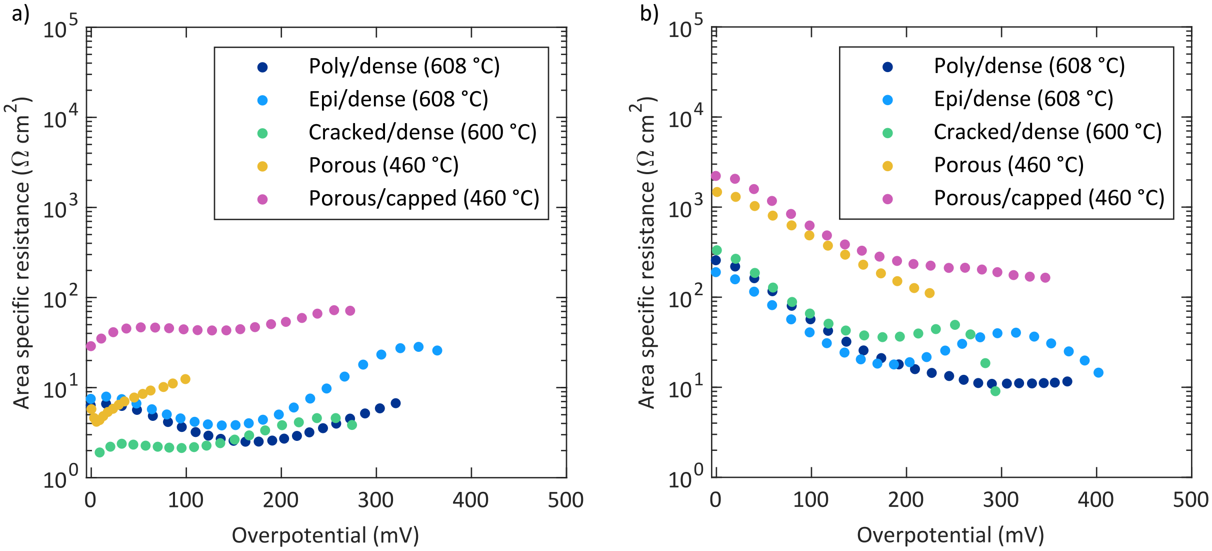

Figure S1: Surface exchange resistance R_s as a function of the electrode overpotential of pristine (a) and annealed (b) electrodes of all different sample types.

The surface exchange resistances R_s of pristine and annealed electrodes of all investigated sample types are depicted in Figure S1 as a function of the respective electrode overpotentials. Please note that R_s was determined from fitting with the equivalent circuit shown in Figure 4a. As described in the main text, this fitting procedure yielded reasonable C_{chem} and thus also R_s values as long as the low frequency arc represented the major part of the respective spectrum. Hence, R_s values are not available over the same overpotential range for all different sample types.

In the pristine state almost all electrodes exhibit a slight increase of R_s at low overpotentials which may be explained by a thermally induced degradation[1–3]. At overpotentials >30 mV, R_s decreases or is almost constant, indicating that apart from the thermal degradation there is a bias related activation, in accordance with literature[4]. Interestingly, at high overpotentials (>150 mV) the resistance increases again. The behavior of the pristine porous electrode differs from the other sample types, which could be caused by crystallisation effects. Please note that R_s of porous electrodes is much lower ($R_s < 0.1\,\Omega\text{cm}^2$) when measured at the

same temperature as dense electrodes (608 °C). For all sample types the R_s values at open circuit conditions increase by several orders of magnitude after annealing for several hours in synthetic air. The surface exchange resistances of all annealed electrodes decrease with increasing overpotential up to about 175 mV. At higher overpotentials, R_s curves increase or stay almost constant.

2 Chemical capacitance during and after long-term annealing

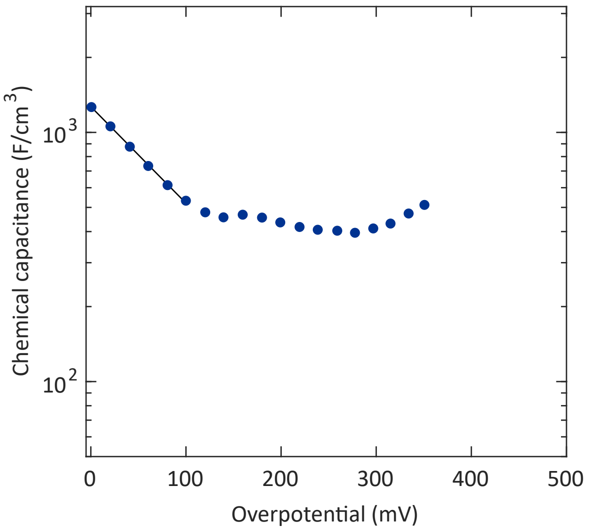

Figure S2: Chemical capacitance of a poly/dense electrode after annealing for 507 h at 608 °C and corresponding fits (solid line).

Figure S2 displays the chemical capacitance of a poly/dense electrode which was annealed for 507 h at 608 °C prior to the measurement. There is no distinct chemical capacitance peak visible after this long annealing time. Figure S3 shows the chemical capacitance and the respective overpotential of a poly/dense electrode as a function of time while a constant application of $U_{DC} = 200$ mV. At the beginning there is a decrease of the chemical capacitance due to an increase of the corresponding overpotential. In the course of the measurement

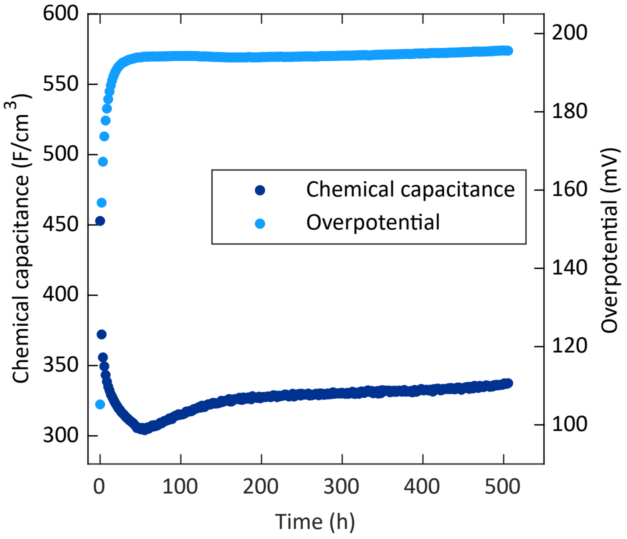

Figure S3: Chemical capacitance and overpotential of a poly/dense electrode over time while applying anodic bias voltage of $U_{DC} = 200\,\text{mV}$ at 608 °C.

the overpotential levels off at about 195 mV. The chemical capacitance shows no significant increase and remains almost constant after 150 h.

3 *In situ* near ambient pressure X-ray photoelectron spectroscopy

Figure S4 shows the setup used for the *in situ* near ambient pressure X-ray photoelectron spectroscopy (NAP-XPS) measurements. The onset of the chemical capacitance peak of an annealed porous electrode is shifted by about 80 mV due to the different atmospheres in the *ex situ* (syn. air) and *in situ* NAP-XPS measurement ($p_{O_2}^{at}$ =1 mbar), see Figure S5a. This is in line with the value calculated from Nernst's equation (84 mV).

The O 1s spectra depicted in Figure S5b were fitted to three components representing "bulk" oxygen (528.5 eV) and a surface-related oxygen component ((531.5 eV), denoted as O 1s surf). The third fitting component is not a chemically distinct oxygen species, but is rather needed to parametrise the asymmetric nature of the O 1s "bulk" signal. In literature this third component was discussed either as a second bulk species[5], or as oxygen in the perovskite

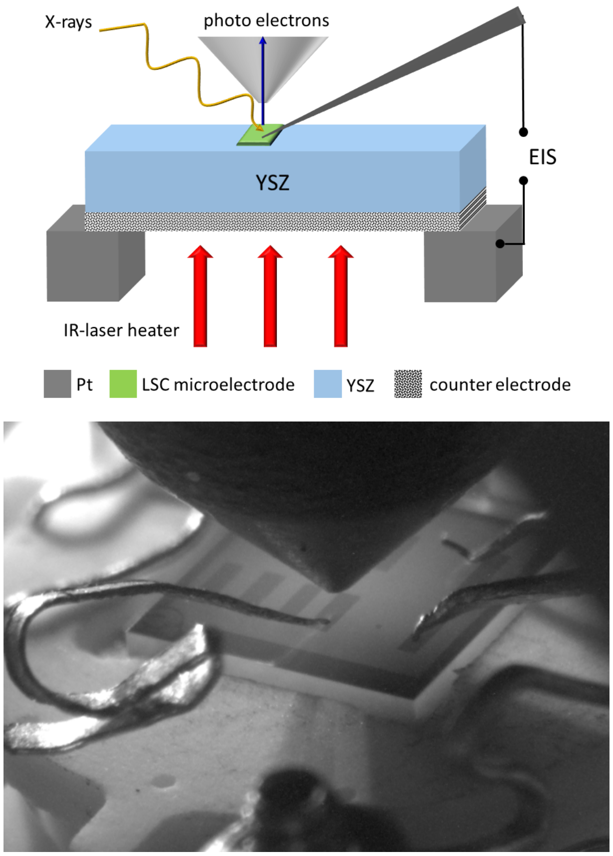

Figure S4: Sketch (top) and picture (bottom) of the NAP-XPS setup used in this study.

termination layer[6]. However, depth profiling by variation of photon energy revealed no significant surface enrichment of this species[5] and it does not seem to change much with the intensity of the S 2p species (see main text). Consequently, we propose a third interpretation: The asymmetric peak shape may be ascribed to the metal-like electronic structure of LSC[7,8]. Since the partly filled conduction band is primarily a hybridization of O 2p and Co 3d states, it is reasonable that the oxygen and cobalt species exhibit a metal-like asymmetry, while the Sr 3d components are symmetric. This interpretation is also in line with the observations of a previous comparative XPS study of different perovskite-type materials.[5] Therein, an asymmetric O 1s bulk peak was only observed for materials with metallic or near-metallic electronic structure.

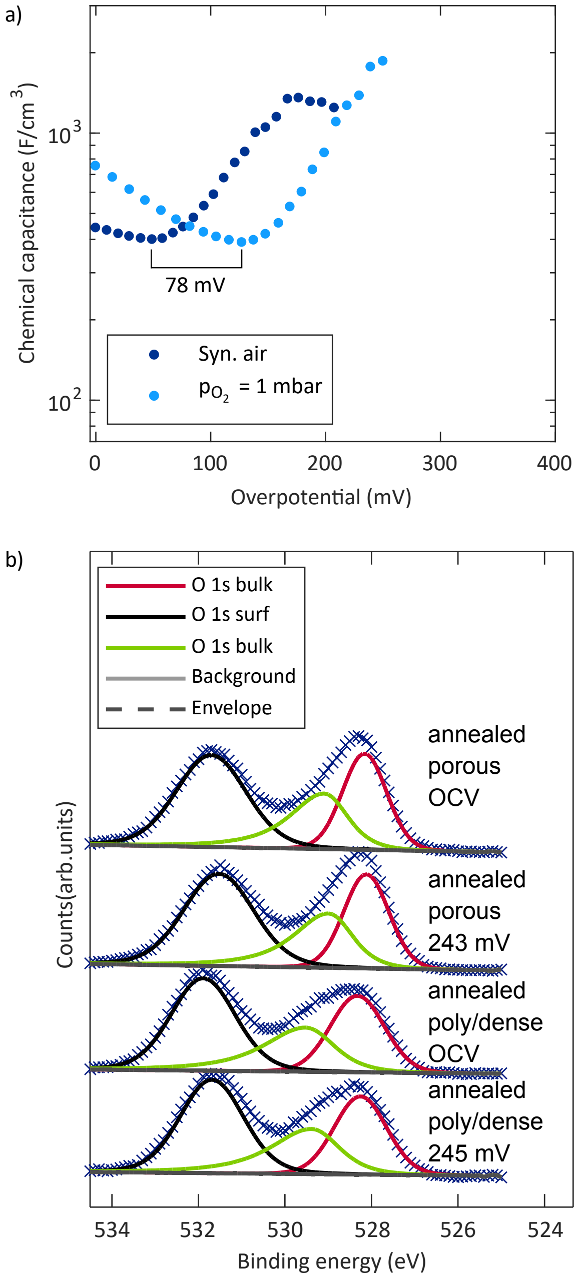

Figure S5: (a) Chemical capacitance of an annealed porous electrode as a function of electrode overpotential, measured *ex situ* at 460 °C in synthetic air and at 1 mbar oxygen partial pressure. (b) O 1s spectra of poly/dense and porous electrodes at OCV and anodic overpotentials at which the chemical capacitance peak occurs (counts marked by blue crosses). Measurements were conducted at 1 mbar oxygen pressure and 460 °C.

4 Transmission electron microscopy

Figure S6 shows a bright field transmission electron microscopy (BF-TEM) image of a closed pore in the bulk of a poly/dense film after applying 750 mV for 1 h.

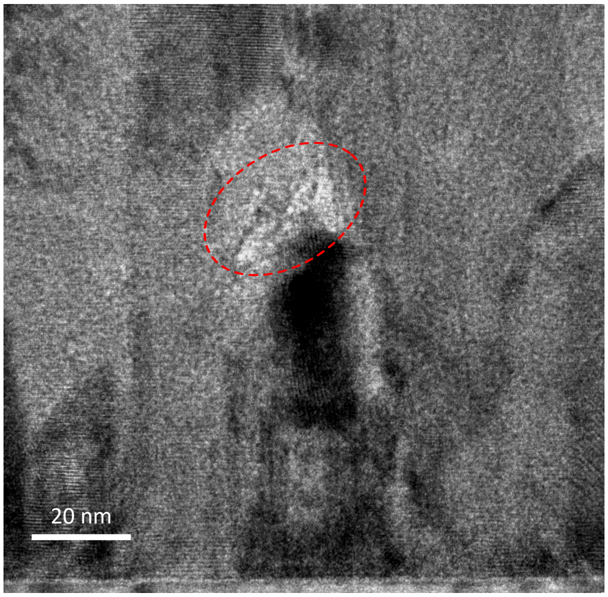

Figure S6: BF-TEM image of a poly/dense electrode after applying 750 mV for 1 h revealing a closed pore in the bulk of the film (marked with dashed red line).

5 Porosity estimation based on a real gas model

In accordance with a recent study[9], we present a detailed model explaining and quantifying the observed chemical capacitance peaks. The build-up of high O_2 gas pressures in closed pores is suggested to cause these capacitive peaks. However, as described in the main text, overpotentials between 150 and 250 mV correspond to effective oxygen partial pressures $p_{O_2}^{WE,eff}$ between 2.8×10^3 and 1.6×10^6 bar. The latter value is far beyond the limits of ideal gas behavior. Therefore, in order to determine the O_2 pressure values in closed pores $p_{O_2}^{pore}$ we have to consider real gas behavior. Here we use the Soave-Redlich-Kwong (SRK) equation

of state[10] to calculate $p_{O_2}^{pore}$:

$$p_{O_2}^{pore} = \frac{RT}{V_{O_2} - b} - \frac{a\alpha}{V_{O_2}(V_{O_2} + b)} = \frac{RT}{\frac{1}{c_{O_2}} - b} - \frac{a\alpha}{\frac{1}{c_{O_2}}(\frac{1}{c_{O_2}} + b)} \quad , \tag{S1}$$

$$a = \frac{0.42747 \cdot R^2 T_c^2}{p_c} \quad , \tag{S2}$$

$$b = \frac{0.08664 \cdot RT_c}{p_c} \quad , \tag{S3}$$

$$\alpha = (1 + (0.480 + 1.574 \cdot \omega_a - 0.176 \cdot \omega_a^2) \cdot (1 - \sqrt{T/T_c}))^2 \quad , \tag{S4}$$

where V_{O_2} and c_{O_2} stand for the molar volume and the concentration of O_2, respectively. R is the universal gas constant and T is the notation for the temperature. Moreover, T_c =154.6 K and p_c =50.46 bar denote the critical temperature and critical pressure of O_2[11], respectively. The temperature dependent term α involves the acentric factor of O_2, *i.e.* $\omega_a = 0.022$, which considers the influence of intermolecular forces depending on the orientation of the molecule.[11] In order to calculate the chemical capacitance of a real gas $C_{chem}^{gas,real}$, we also have to determine the fugacity coefficient ϕ. According to the Soave-Redlich-Kwong (SRK) equation ϕ can be calculated as follows[10]

$$\phi = \frac{f}{p_{O_2}^{pore}} = Z - 1 - \ln\left(Z - \frac{bp_{O_2}^{pore}}{RT}\right) - \frac{a}{bRT} \cdot \ln\left(1 + \frac{bp_{O_2}^{pore}}{ZRT}\right) \quad , \tag{S5}$$

with f being the fugacity and Z denoting the compressibility factor which is defined as

$$Z = \frac{p_{O_2}^{pore}}{c_{O_2} RT} \quad . \tag{S6}$$

Then, the chemical capacitance of the real gas in closed pores can be determined:

$$C_{chem}^{gas,real} = 16F^2\lambda V \cdot \left(\frac{\partial \mu_{O_2}}{\partial c_{O_2}}\right)^{-1} = 16F^2\lambda V \cdot \left(\frac{\partial\left(\mu_{O_2}^{0,T} + RT\ln\left(\frac{\phi p_{O_2}^{pore}}{1\,\mathrm{bar}}\right)\right)}{\partial c_{O_2}}\right)^{-1}$$
$$= \frac{16F^2\lambda V}{RT} \cdot \left(\frac{\partial\left(\ln\left(\frac{\phi p_{O_2}^{pore}}{1\,\mathrm{bar}}\right)\right)}{\partial c_{O_2}}\right)^{-1} , \tag{S7}$$

where λ denotes the film porosity, *i.e.* the volume fraction of closed pores with respect to the entire film volume V. With the values for $p_{O_2}^{pore}$ and ϕ obtained from Equations S1 and S5, respectively, $\frac{\partial \mu_{O_2}}{\partial c_{O_2}}$ in Equation S7 was calculated numerically. To be able to fit our experimental data to Equation S7, the overpotential in the working electrode η_{WE} with respect to the oxygen partial pressure in synthetic air is related to the fugacity of the real gas equation as follows

$$\eta_{WE} = \frac{RT}{4F} \cdot \ln\left(\frac{f}{0.21\,\mathrm{bar}}\right) = \frac{RT}{4F} \cdot \ln\left(\frac{\phi p_{O_2}^{pore}}{0.21\,\mathrm{bar}}\right) . \tag{S8}$$

Figure S7 shows the experimentally obtained volume specific chemical capacitance of a poly/dense electrode after applying $U_{DC} = 1000\,\mathrm{mV}$ for 1 h at 608 °C. In order to fit this experimental data, we also have to consider the defect-related chemical capacitance C_{chem}^{defect}, which is predominant at low overpotentials. As described in the main text, this contribution can be calculated as follows

$$C_{chem} \approx \frac{4F^2Vc_V^{at}}{RT} \cdot \exp\left(\frac{-\alpha F\eta_{WE}}{RT}\right) . \tag{S9}$$

A corresponding fit of the experimental chemical capacitance data up to the minimum at about 100 mV yields an exponential factor of $\alpha = 0.56$. This fit and the associated extrapolation to higher overpotentials is shown in Figure S7. Additionally, the capacitance

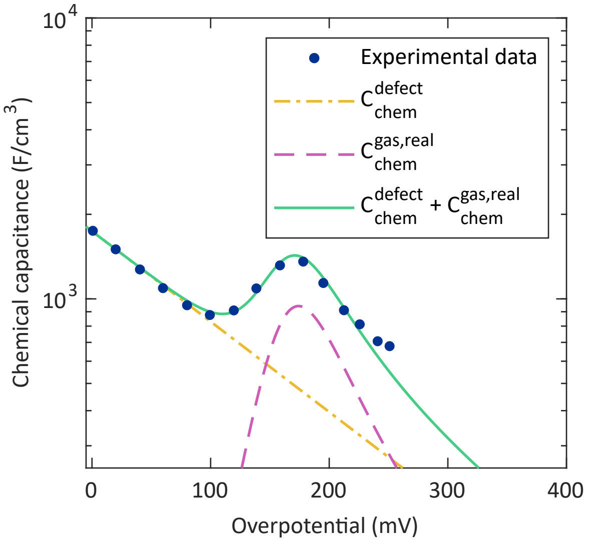

Figure S7: Experimental chemical capacitance data of a poly/dense electrode after applying $U_{DC} = 1000\,\mathrm{mV}$ for 1 h at 608 °C. The orange dash-dotted line illustrates the fit and the corresponding extrapolation of the defect-related chemical capacitance C_{chem}^{defect}. The purple dashed line represents the chemical capacitance of O_2 in closed pores $C_{chem}^{gas,real}$ according to the real gas model of Equation S7. The green solid line shows the sum of the defect-related extrapolation and the capacitance of the real gas.

contribution of O_2 in closed pores $C_{chem}^{gas,real}$, which was calculated according to Equation S7, is depicted in Figure S7 (purple dashed line). The corresponding porosity value $\lambda = 0.0062$ for $C_{chem}^{gas,real}$ was determined from a least-squares optimization of the sum of the two capacitance contributions, *i.e.* $C_{chem} = C_{chem}^{defect} + C_{chem}^{gas,real}$ (green solid line in Figure S7), to the experimental capacitance data.

References

(1) Kubicek, M.; Limbeck, A.; Frömling, T.; Hutter, H.; Fleig, J. Relationship between Cation Segregation and the Electrochemical Oxygen Reduction Kinetics of $La_{0.6}Sr_{0.4}CoO_{3-\delta}$ Thin Film Electrodes. *Journal of The Electrochemical Society* **2011**, *158*, B727.

(2) Rupp, G. M.; Téllez, H.; Druce, J.; Limbeck, A.; Ishihara, T.; Kilner, J.; Fleig, J.

Surface chemistry of $La_{0.6}Sr_{0.4}CoO_{3-\delta}$ thin films and its impact on the oxygen surface exchange resistance. *J Mater Chem A* **2015**, *3*, 22759–22769.

(3) Cai, Z.; Kubicek, M.; Fleig, J.; Yildiz, B. Chemical Heterogeneities on $La_{0.6}Sr_{0.4}CoO_{3-\delta}$ Thin Films—Correlations to Cathode Surface Activity and Stability. *Chemistry of Materials* **2012**, *24*, 1116–1127.

(4) Baumann, F. S.; Fleig, J.; Habermaier, H.-U.; Maier, J. Impedance spectroscopic study on well-defined (La,Sr)(Co,Fe)$O_{3-\delta}$ model electrodes. *Solid State Ionics* **2006**, *177*, 1071–1081.

(5) Nenning, A.; Opitz, A. K.; Rameshan, C.; Rameshan, R.; Blume, R.; Hävecker, M.; Knop-Gericke, A.; Rupprechter, G.; Klötzer, B.; Fleig, J. Ambient Pressure XPS Study of Mixed Conducting Perovskite-Type SOFC Cathode and Anode Materials under Well-Defined Electrochemical Polarization. *The Journal of Physical Chemistry C* **2016**, *120*, 1461–1471.

(6) Crumlin, E. J.; Mutoro, E.; Hong, W. T.; Biegalski, M. D.; Christen, H. M.; Liu, Z.; Bluhm, H.; Shao-Horn, Y. In Situ Ambient Pressure X-ray Photoelectron Spectroscopy of Cobalt Perovskite Surfaces under Cathodic Polarization at High Temperatures. *The Journal of Physical Chemistry C* **2013**, *117*, 16087–16094.

(7) Mizusaki, J.; Mima, Y.; Yamauchi, S.; Fueki, K.; Tagawa, H. Nonstoichiometry of the perovskite-type oxides $La_{1-x}Sr_xCoO_{3-\delta}$. *Journal of Solid State Chemistry* **1989**, *80*, 102–111.

(8) Kuhn, M.; Hashimoto, S.; Sato, K.; Yashiro, K.; Mizusaki, J. Oxygen nonstoichiometry and thermo-chemical stability of $La_{0.6}Sr_{0.4}CoO_{3-\delta}$. *Journal of Solid State Chemistry* **2013**, *197*, 38–45.

(9) Krammer, M.; Schmid, A.; Siebenhofer, M.; Bumberger, A. E.; Herzig, C.; Limbeck, A.; Kubicek, M.; Fleig, J. Formation and Detection of High-Pressure Oxygen in Closed

Pores of $La_{0.6}Sr_{0.4}CoO_{3-\delta}$ Solid Oxide Electrolysis Anodes. *ACS Applied Energy Materials* **2022**, *5*, 8324–8335.

(10) Soave, G. Equilibrium constants from a modified Redlich-Kwong equation of state. *Chemical Engineering Science* **1972**, *27*, 1197–1203.

(11) Kleiber, M.; Joh, R. *Properties of Pure Fluid Substances in VDI Heat Atlas*, 2nd ed.; Springer Berlin Heidelberg, 2010; Chapter Properties of Pure Fluid Substances, pp 301–393.
